# Macrophage‐Targeted Magnesium Ion‐Nourisher for NLRP3 Inflammasome Inhibition to Enhance Liver Inflammatory Disease Treatment

**DOI:** 10.1002/advs.202513798

**Published:** 2025-11-05

**Authors:** Li Wang, Zhuo Yan, Sindhu Yalavarthi, Yusif Abdul‐Rashid, Kiki Parker, Kyle Nowlin, Ethan Li, Jordan Mack, Josephine Wei, Hunter Vu, Zhenquan Jia, Jianjun Wei, Jilong Wang, Kerui Wu

**Affiliations:** ^1^ Department of Nanoscience Joint School of Nanoscience and Nanoengineering University of North Carolina at Greensboro Greensboro NC 27401 USA; ^2^ Joint Centre of Translational Medicine The First Affiliated Hospital of Wenzhou Medical University Wenzhou Medical University Wenzhou Zhejiang 325000 China; ^3^ Department of Nanoengineering North Carolina Agricultural and Technical State University Greensboro NC 27401 USA; ^4^ Department of Biology University of North Carolina at Greensboro Greensboro NC 27412 USA; ^5^ J. Crayton Pruitt Family Department of Biomedical Engineering University of Florida Gainesville FL 32611 USA

**Keywords:** inflammation, macrophage, magnesium nano‐nourisher, NLRP3 inflammasome, stem cell‐derived vesicles

## Abstract

Magnesium (Mg) exerts important functions in immune regulation. Fluctuations of Mg levels significantly impact immune cell behavior, such as differentiation and inflammatory phenotypes of macrophages. However, exploiting Mg as an immunomodulatory intervention is challenging due to its unclear mechanism and broad impact across diverse cells. To overcome this, a nanosized Mg ion‐nourisher is engineered, termed MgC^@PS^, that enables efficient macrophage‐targeted intracellular Mg^2^⁺ (iMg^2+^) delivery by exploiting macrophages’ efferocytosis in response to phosphatidylserine. It is found that targeted enrichment of magnesium ions (Mg^2^⁺) into macrophages effectively inhibits NOD‐like receptor pyrin domain containing 3 (NLRP3) inflammasome activation‐induced pyroptosis, and alleviates hyperactive inflammatory responses. Single‐cell RNA sequencing reveals fortified resilience of Kupffer cells from pyroptosis and upregulation of antioxidant gene expression after MgC^@PS^ treatment. Moreover, by incorporating stem cell components into the MgC^@PS^, the integrity of the intestinal barrier, addressing the barrier leakage commonly observed in the gut‐liver axis. These findings demonstrate the pivotal role of iMg^2^⁺ in mitigating macrophage‐mediated liver‐gut inflammation. Targeted delivery of Mg to macrophages emerges as a promising strategy to inhibit excessive inflammation and promote tissue recovery.

## Graphical abstract

This report describes a nano magnesium nourisher, named MgC^@PS^, capable of targeted Mg^2^⁺ delivery to macrophages, effectively suppressing The nuclear factor kappa B (NF‐κB) activation and mitigating NLRP3 inflammasome‐driven pyroptosis in liver inflammation. Integrated with stem cell vesicle encapsulation, MgC^@PS_SCV^ safeguards Kupffer cells, alleviates oxidative stress, reduces inflammatory monocyte infiltration, and accelerates tissue repair, presenting a promising approach for managing gut‐liver inflammatory disorders.

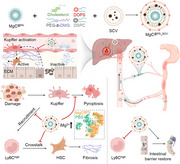

## 1 Introduction

Nutritional metal ions are essential micronutrients that play critical roles in maintaining physiological homeostasis and modulating immune responses.^[^ ^1^, ^2^, ^3^, ^4^ ^]^ Magnesium ion (Mg^2^⁺) as the most abundant intracellular divalent metal ion in eukaryotic cells is essential for numerous biological processes.^[^ ^5^, ^6^, ^7^ ^]^ Interestingly, it is also found to be a critical factor influencing chronic inflammation.^[^ ^8^ ^]^ Epidemiologic evidences suggest an inverse correlation between magnesium intake and the risk of inflammatory diseases.^[^ ^9^ ^]^ Hypomagnesemia can be commonly observed in patients with inflammatory diseases, such as non‐alcoholic steatohepatitis (NASH) and Crohn's disease, and magnesium deficiency is found to be tightly associated with the pathogenesis.^[^ ^10^, ^11^, ^12^ ^]^ Mechanistically, iMg^2+^ level is meticulously regulated, and fluctuations of iMg^2^⁺ in immune cells can critically impact their metabolism and function, leading to substantial changes in inflammatory responses.^[^ ^13^, ^14^, ^15^ ^]^ Recent studies highlighted the role of Mg^2^⁺ in modulating immune cell function, particularly the macrophages, which indispensably governs inflammation development. Mg^2^⁺ has been found to have anti‐inflammatory effect in macrophages and microglial cells, and its deficiency exacerbates inflammatory responses.^[^ ^16^, ^17^, ^18^ ^]^ With such strong evidence suggesting the anti‐inflammatory role of Mg^2^⁺, magnesium supplementation has been tested in clinical trials as a therapy for different inflammatory diseases.^[^ ^19^, ^20^ ^]^ However, these clinical trials yield mixed results. One of the major issues with the traditional oral or intravenous magnesium supplementation is its failure to sustain therapeutic Mg^2^⁺ levels in the immune cells, driving inflammation. In vitro testing verified that an extracellular Mg^2^⁺ concentration of more than 5 mM is needed to induce anti‐inflammatory phenotype in macrophages.^[^ ^21^ ^]^ Systemic administration of Mg^2^⁺ at this level can cause severe hypermagnesemia, which may result in adverse effects on muscle cell, neurons, cardiomyocytes, and renal cells, etc. and cause severe symptoms such as heart block and coma.^[^ ^22^, ^23^ ^]^ Prior clinical attempts to alleviate inflammation through increased concentrations of extracellular Mg^2^⁺ have shown limited efficacy, likely due to the inefficient regulation of intracellular iMg^2+^ level and off‐target distribution.^[^ ^24^, ^25^, ^26^ ^]^ Thus, achieving macrophage‐targeted intracellular delivery of Mg^2^⁺ and elucidating its anti‐inflammatory mechanisms are of paramount importance.

Upon aberrant stimulation, macrophages, as the first defensive line, initiate an inflammatory reaction, such as pyroptosis and cGAS‐STING activation, to combat threats.^[^ ^27^, ^28^, ^29^, ^30^, ^31^ ^]^ However, excessive activation of these inflammatory pathways can cause severe tissue damage and even life‐threatening systemic inflammation.^[^ ^32^, ^33^, ^34^ ^]^ Among these, NLRP3 inflammasome plays a pivotal role in amplifying macrophage‐driven inflammation by inducing pyroptosis and recruiting circulating monocytes.^[^ ^35^, ^36^, ^37^, ^38^, ^39^ ^]^ In liver injury, environmental stressors such as oxidative stress and mitochondrial dysfunction trigger NLRP3 activation, driving macrophage differentiation into pro‐fibrotic phenotypes that exacerbate hepatic stellate cell (HSC)‐mediated fibrosis.^[^ ^40^, ^41^, ^42^, ^43^ ^]^ Moreover, the liver and gut are closely interconnected through the gut‐liver axis, where liver injury disrupts immune homeostasis and compromises intestinal barrier integrity.^[^ ^44^, ^45^, ^46^, ^47^ ^]^ This dysfunction allows gut‐derived endotoxins and microbial metabolites to enter the liver via the portal vein, perpetuating hepatic inflammation.^[^ ^48^, ^49^ ^]^ Notably, Mg^2^⁺ deficiency can worsen gut dysbiosis and impair mucosal immunity, thereby amplifying inflammation through this axis.^[^ ^10^, ^50^, ^51^ ^]^ Given the central role of macrophage‐mediated inflammation in both hepatic and intestinal injury, precisely targeting macrophages to suppress NLRP3 inflammasome activation while replenishing Mg^2^⁺ emerges as a promising strategy to interrupt this pathological crosstalk.

**Scheme 1:**
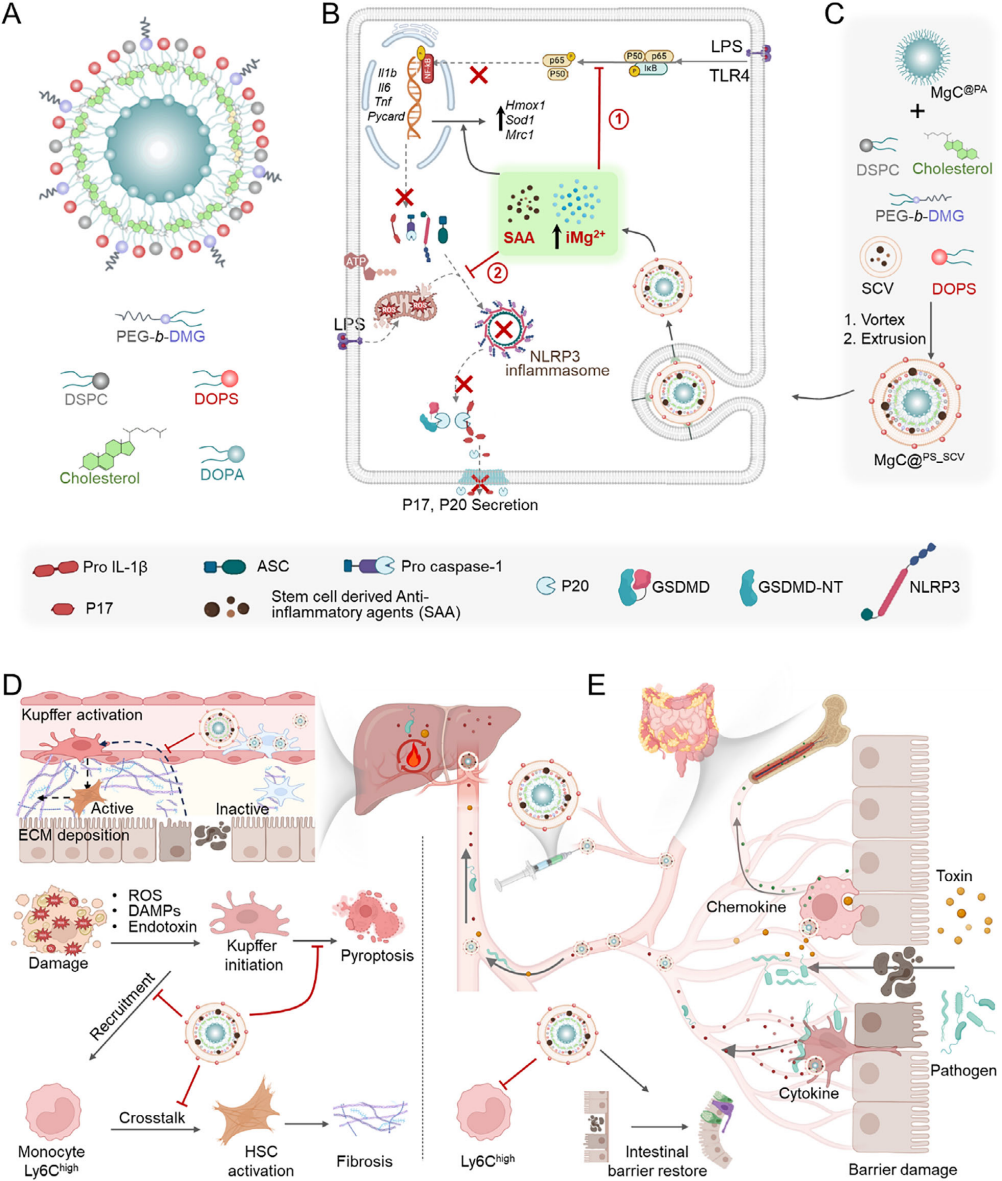
Schematic showing treatment mechanisms of nano magnesium nourisher (MgC^@PS_SCV^) for liver injury and ulcerative colitis (UC). A) Schematic showing the structural composition of MgC^@PS^. MgC^@PA^ was prepared in a water‐in‐oil method with DOPA on the surface. DOPS, Cholesterol, and DMG‐PEG_2000_ were incorporated into the outer layer by hydration. B) Illustration of MgC^@PS_SCV^ preparation by MgC^@PA^ and stem cell vesicle co‐extrusion with DOPS addition. C) During inflammation, ATP, ROS, pathogen, and toxin will stimulate macrophages and cause NLRP3 inflammasome formation, which further cleaves IL‐1β for an inflammatory reaction. MgC^@PS_SCV^ internalization and dissociation elevated intracellular Mg^2+^ (iMg^2+^) level and inhibited P65 activation, which suppressed the inflammation process. MgC^@PS_SCV^ also showed NLRP3 inflammasome formation after lipopolysaccharide (LPS) priming. D,E) MgC^@PS_SCV^ demonstrated effective performance in treating liver injury and UC by inhibiting monocyte differentiation into pro‐inflammatory phenotype, protecting tissue resident macrophage population from pyroptosis, and enhancing intestinal barrier recovery (Created in https://biorender.com/).

Here, we identified Mg^2^⁺ as a potent regulator for macrophage‐driven inflammation treatment. We discovered that targeted intracellular enrichment of Mg^2^⁺ effectively suppresses inflammatory pathway activation, particularly through NLRP3 pathway inhibition. To achieve macrophage‐specific delivery, we designed an efferocytosis‐inspired nano Mg ion‐nourisher, termed MgC^@PS^, that is coated with dioleoyl phosphatidylserine (DOPS) to enable macrophage‐targeted internalization and rapid intracellular Mg^2^⁺ accumulation (**Scheme** **1**A).^[^ ^52^ ^]^ In the CCl_4_‐induced liver injury model, MgC^@PS^ treatment suppressed NLRP3 inflammasome activation and improved the survival of resident macrophages (Scheme 1B), attenuating the fibrotic crosstalk between macrophages and hepatic stellate cells (HSCs) (Scheme 1D). Single‐cell RNA sequencing (scRNA‐seq) further revealed enhanced Kupffer cell survival and upregulated antioxidant gene expression, providing molecular evidence of Mg^2^⁺ enrichment caused benefits. Building on these findings, we integrated stem cell vesicles (SCVs), endowed with the potent immunoregulatory and regenerative effects through diverse biomolecules, such as growth factors, cytokines, and microRNAs^[^ ^53^, ^54^, ^55^ ^]^ into our MgC^@PS^ platform. This integration resulted in the development of a hybrid nano Mg nourisher (MgC^@PS_SCV^) was designed to treat co‐existing liver‐gut inflammation in a dual‐disease mouse model (CCl_4_‐induced liver injury and dextran sulfate sodium (DSS)‐induced colitis) (Scheme 1C). MgC^@PS_SCV^ treatment significantly suppressed inflammatory monocyte infiltration and alleviated inflammation (Scheme 1E), providing a macrophage‐targeted, anti‐inflammatory hybrid nano magnesium nourisher for treating inflammatory diseases.

## 2 Results and Discussion

### 2.1 Targeted Intracellular Delivery of MgC^@PS^ to Macrophage in Inflammatory Liver Tissues and Colon Tissues

**Figure 1:**
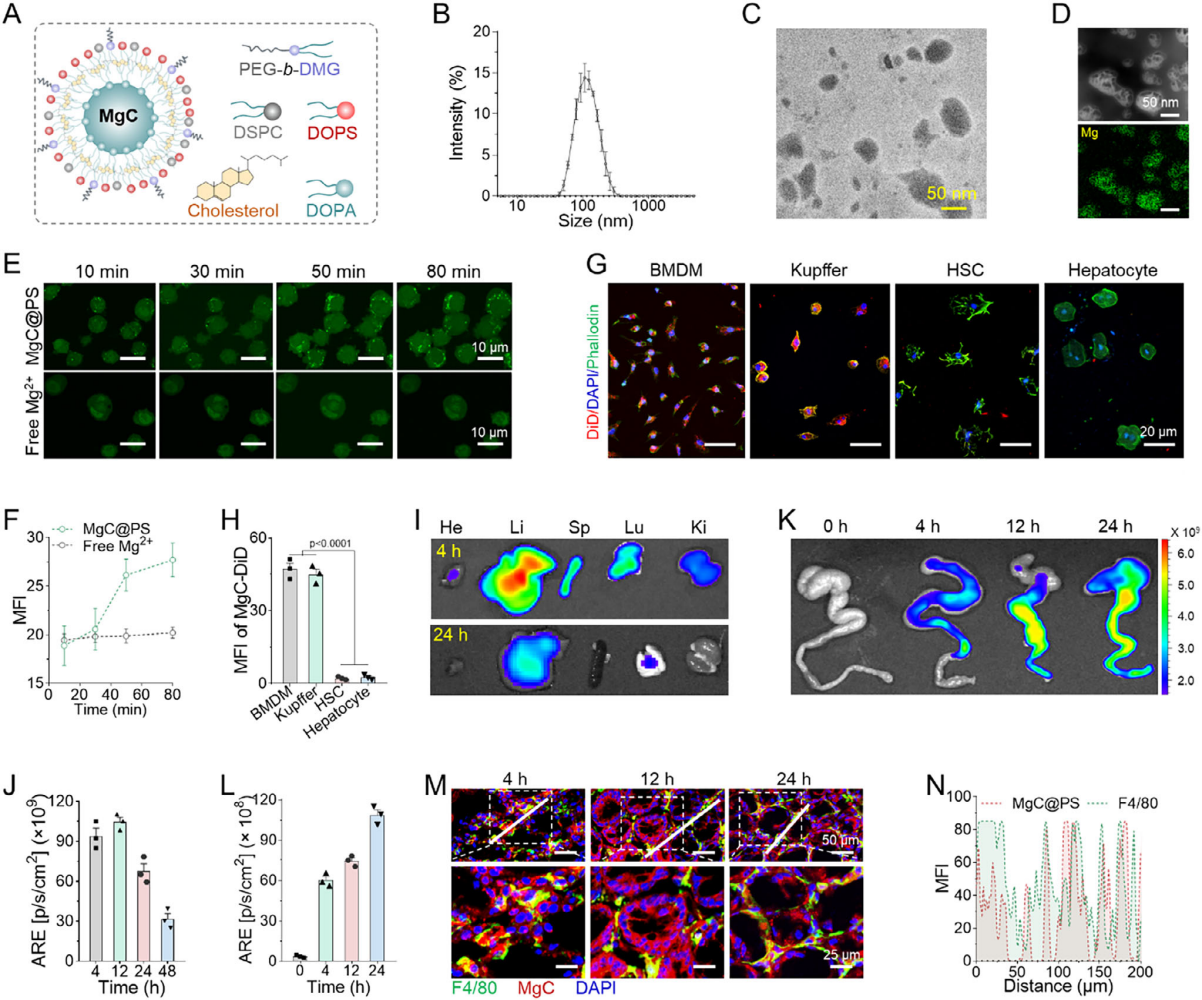
MgC^@PS^ efferocytosis‐like targeted enrichment to macrophages in inflammatory liver tissues and colon tissues. A) Scheme of MgC structure of the core and the composition of the lipid bilayer. B) DLS measurements of MgC^@PS^. C) Typical TEM images of MgC^@PS^. D) Element mapping of Mg content in MgC^@PS^ nanoparticles. E) Time‐dependent fluorescent images visualizing the intracellular Mg^2+^ level of THP‐1 cells during MgC^@PS^ incubation, Mg^2+^ was indicated by a fluorescent probe, Magnesium Green^AM^. F) MFI calculation of Mg^2+^ fluorescence in THP‐1 cells by ImageJ (*n* = 3). G) Internalization of MgC^@PS^ in different primary cells isolated from C57BL6 mice, including BMDMs, Kupffer cells, HSCs, and hepatocytes. H) Calculation of mean fluorescence intensity (MFI) of MgC^@PS^ in different primary cells according to panel E by imageJ (*n* = 3). I) In vivo optical imaging system (IVIS) showing in vivo distribution of MgC^@PS^ in main organs after 4, 24 h post intravenous (i.v.) injection. J) Calculation of time‐dependent liver fluorescence from IVIS images, data related to Figure 1K (*n* = 3). K) Typical IVIS images of 3% DSS‐induced inflammatory mouse colon tissues after MgC^@PS/DiD^ administration. L) Calculation of time‐dependent colon fluorescence from IVIS images, data related to Figure 1M (*n* = 3). M) Typical confocal laser scanning microscopy (CLSM) images of colon tissue after i.v. administration of MgC^@PS/DiD^. Macrophages were labeled with anti‐F4/80 mAb (Green), cell nuclei were stained with DAPI in blue, and the red signal indicates MgC^@PS/DiD^ nanoparticle. N) Fluorescence distribution profile of MgC^@PS/DiD^ and macrophage along the solid white lines. The overlap area indicates the macrophage internalization of MgC^@PS/DiD^. Data are presented as mean ± S.D.

To investigate the performance of nutritional metal ions in modulating macrophage inflammation, we synthesized various metal ion nanoparticles (Mg, Zn, Ca) with DOPS on their surface (**Figure** **1**A,B; Figure S1A, Supporting Information). All nanoparticles exhibited similar sizes ranging from 108.3 to 136.7 nm with narrow size distributions (PDI range, 0.17–0.24) (Figure S1A, Supporting Information). The incorporation of DOPS conferred a negative surface charge to all metal nanoparticles. After treating LPS‐stimulated macrophages with these nanoparticles concurrently, we evaluated the inflammatory cytokine (IL‐1β, IL‐6) transcription level and inflammatory marker expression, inducible nitric oxide synthase (iNOS). We observed that Zn and Mg nanoparticles demonstrated anti‐inflammatory effects, whereas Ca nanoparticles showed no anti‐inflammatory activity (Figure 1B). Among the Zn and Mg groups, MgC^@PS^ treatment exhibited the strongest effect, reducing iNOS expression by 42% (Figure S1C, Supporting Information). TEM images confirmed a similar particle size and successful incorporation of Mg into MgC^@PS^ by element mapping (Figure 1C,D). Protected by bilayer lipid, MgC^@PS^ demonstrated high stability in physiologically relevant 10% FBS (Figure S1D,E, Supporting Information). To determine whether the anti‐inflammatory effect of MgC^@PS^ is due to its internalization and subsequent Mg^2^⁺ release, we monitored intracellular Mg^2^⁺ (iMg^2^⁺) levels using the Mg^2^⁺‐specific fluorescent probe, Magnesium Green, AM. During the incubation, MgC^@PS^ treatment caused a significant increase in iMg^2^⁺ levels, whereas free Mg^2^⁺ treatment showed no fluctuation in iMg^2^⁺ levels (Figure 1E,F). These results indicate that MgC^@PS^ facilitates a rapid increase in iMg^2^⁺ level within macrophages compared to free Mg^2^⁺. To evaluate the efferocytosis‐mimicking behavior of MgC^@PS^ induced by DOPS incorporation, we tested its internalization in various cell lines. DiD lipophilic dye was incorporated into the lipid layer of MgC^@PS^ to visualize nanoparticle micro‐distribution. Both RAW264.7 and THP‐1 macrophage cell lines exhibited rapid phagocytosis within 45 min of incubation, while HUVECs and 4T1 cells demonstrated limited internalization. This preferred uptake is due to DOPS on the nanoparticle surface that macrophages recognize as debris from ruptured cells (Figure S2A; Figure S2B,C, Supporting Information).^[^ ^56^ ^]^ Compared to macrophages, bone marrow‐derived dendritic cells showed moderate internalization after 1 h incubation. HL60 cell, a neutrophil‐like cell line, showed almost no uptake even after 4 h internalization (Figure S2C, Supporting Information). We further assessed MgC^@PS^ internalization in primary innate immune cells, including bone marrow‐derived macrophages (BMDMs) and Kupffer cells, as well as HSCs and hepatocytes. BMDMs and Kupffer cells showed rapid phagocytosis of MgC^@PS^, whereas HSCs and hepatocytes exhibited limited uptake (Figure 1G,H). These findings provide evidence of the efferocytosis‐mimicking properties of MgC^@PS^. Next, we analyzed the in vivo distribution of MgC^@PS^ following intravenous (i.v.) administration in CCl_4_‐ and DSS‐treated C57BL/6 mice. In the CCl_4_‐induced liver injury model, MgC^@PS^ predominantly accumulated in liver tissues, with significant retention even after 24 h (Figure 1I,J; Figure S2D, Supporting Information). Due to the close communication between the gut and liver, inflammatory bowel disease showed a high correlation with liver inflammatory disease progression.^[^ ^44^ ^]^ Thus, we established a DSS‐induced ulcerative colitis (UC) model to evaluate MgC^@PS^ biodistribution. MgC^@PS^ demonstrated significant preferred accumulation in inflamed colon tissues as early as 4 h post‐injection, with increasing enrichment over 24 h (Figure 1K,L). By analyzing MgC^@PS^ fluorescence colocalization with F4/80⁺ macrophages, we confirmed the targeted delivery of MgC^@PS^ to macrophages in the inflamed colon (Figure 1M,N; Figure S2E–G, Supporting Information). These results collectively demonstrated that MgC^@PS^ exhibits macrophage‐specific targeting and efficient intracellular Mg^2^⁺ delivery, supporting its therapeutic potential in inflammatory conditions. After digestion of colon tissues, a single cell suspension was analyzed by flow cytometry (FC) to evaluate the monocyte uptake. To further analyze the preferred uptake of MgC^@PS^ in vivo, MgC^@PS/DiL^ was i.v. The administered and colon tissues were excised for flow cytometry analysis. MgC^@PS/DiL^ treatment revealed a high proportion of Ly6C+ cells within the CD45+ MgC^@PS/DiL^+ population, whereas neutrophils exhibited negligible uptake (Figure S3, Supporting Information), aligning with our in vitro internalization findings.

### 2.2 MgC^@PS^ Treatment Effectively Protected Resident Macrophages with Tolerogenic Phenotype

**Figure 2:**
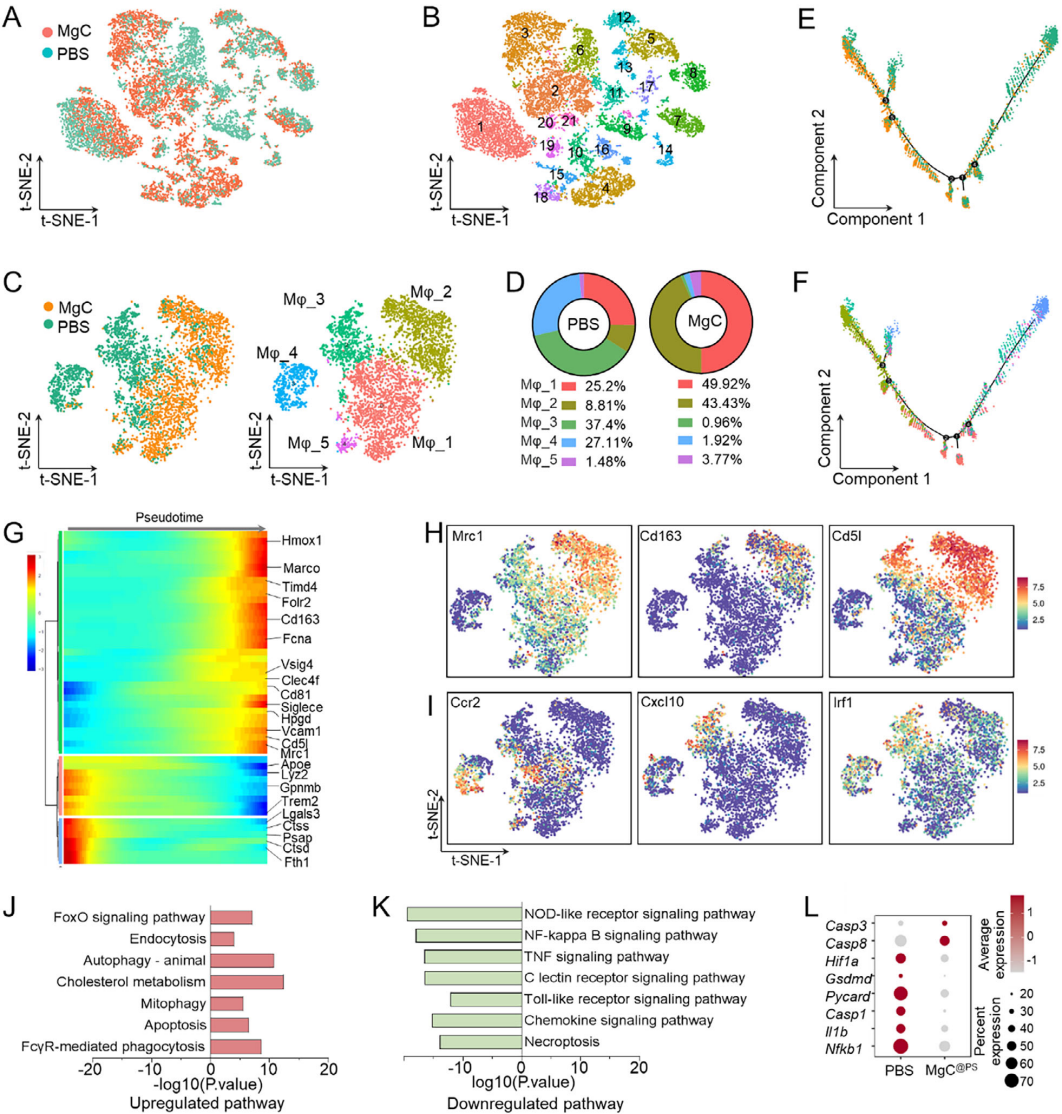
Single‐cell transcriptomics analysis of non‐parenchymal cells to investigate genes and key molecular pathways modulated by MgC^@PS^ treatment in CCl_4_‐induced inflammation and fibrosis. A) t‐SNE projections of graph‐based cell clusters identified from scRNA‐seq data of hepatic CD45⁺ cells from CCl_4_‐treated mice and MgC^@PS^+CCl_4_‐treated mice. Each point represents a single cell, color‐coded by sub‐cluster assignment. B) Cluster map showing the assigned identities of each cluster defined in panel A. Cluster identities include: B cells (1), macrophages (2, 3, 6, 9, 20, 21), dendritic cells (7, 11, 19), endothelial cells (4, 10, 15, 16), T cells (5, 12, 13), natural killer (NK) cells (8), neutrophils (14), proliferative lymphocytes (17), and fibroblasts (18). C) Subclusters of five macrophage phenotypes. t‐SNE visualization highlights the multiple macrophage subpopulations identified in both MgC^@PS^‐treated and control groups. Figure S9 (Supporting Information) provides a heatmap of the top ten differentially expressed genes for each subcluster. D) Pie chart showing the proportions of the five macrophage subpopulations among all macrophages in control and MgC^@PS^‐treated mice. E) Pseudotime trajectory of macrophages, colored by treatment groups (PBS versus MgC^@PS^). F) Pseudotime trajectory showing macrophage subclusters (cluster_1 to cluster_5) and their developmental trajectories. G) Heatmap illustrating differentially expressed genes along the pseudotime trajectory, revealing a transition from a pro‐inflammatory to an anti‐inflammatory phenotype. H) t‐SNE plot showing the relative distribution of anti‐inflammatory markers. I) t‐SNE plot showing the relative distribution of pro‐inflammatory markers. J,K) Selected significantly enriched pathways from KEGG analysis of DEGs in CD45⁺, F4/80⁺ macrophages. Panel J highlights upregulated pathways, while panel K shows downregulated pathways in MgC^@PS^‐treated mice. L) Heatmaps of DEGs across macrophage subclusters, showing distinct expression patterns from scRNA‐seq data.

To investigate immune cell heterogeneity following MgC^@PS^ treatment, we performed scRNA‐seq on immune cells isolated from control mice treated with CCl_4_ and those treated with both CCl_4_ and MgC^@PS^. After quality control and filtering, we identified, clustered, and visualized the most differentially expressed transcripts in each immune cell cluster. t‐Distributed Stochastic Neighbor Embedding (t‐SNE) plots were generated by Cell Ranger, the official analysis software from 10× Genomics for precise cell identity assignment. A total of 21 clusters were identified based on t‐SNE dimensionality reduction (**Figure** **2**A,B). Further analysis focused on five macrophage subclusters determined through unbiased cell‐type recognition (Figure 2C) using specific marker genes outlined in Figure S4A (Supporting Information). Proportional analysis revealed that Mφ_3 and Mφ_4 were the dominant macrophage populations in control mice treated with CCl_4_ alone (PBS), whereas Mφ_1 and Mφ_2 predominated in the liver tissues of MgC^@PS^‐treated mice (Figure 2D). Notably, MgC^@PS^ treatment significantly increased the proportion of Mφ_2 and reduced the proportion of Mφ_4. To explore the developmental trajectory of macrophages following MgC^@PS^ treatment, we conducted pseudotime analysis (Figure 2E). Homeostatic Mφ_1 macrophage transitioned toward Mφ_3 and Mφ_4 phenotypes after CCl_4_ treatment. However, MgC^@PS^ treatment reprogrammed Mφ_3 and Mφ_4 macrophages into Mφ_2, an anti‐inflammatory subcluster evidenced by high *Mrc1* and *Cd163* mRNA levels (Figure 2F,H). A transcriptome trajectory heatmap further demonstrated a shift in macrophages from a pro‐inflammatory to an anti‐inflammatory phenotype following MgC^@PS^ treatment (Figure 2G). To visualize the expression of anti‐ and pro‐inflammatory markers, we applied t‐SNE plot analysis. Key anti‐inflammatory genes, such as *Mrc1*, *Cd163*, *Cd5l*, and *Hmox1*, were highly expressed in the Mφ_2 and Mφ_1 subclusters, which were predominantly derived from MgC^@PS^‐treated mice (Figure 2H; Figure S4B, Supporting Information). Conversely, pro‐inflammatory genes, including *Ccr2*, *Cxcl10*, and *Irf1*, exhibited elevated expression in the Mφ_3 and Mφ_4 populations, which were primarily derived from control mice (Figure 2I; Figure S4C, Supporting Information). During CCl_4_‐induced liver injury, recruited macrophages demonstrated phenotypic plasticity, transitioning to a pro‐inflammatory state in response to damage‐associated environmental signals. To identify the primary pathways affected by MgC^@PS^ treatment, we performed Kyoto Encyclopedia of Genes and Genomes (KEGG) analysis on the macrophage transcriptomes from the liver tissues of MgC^@PS^‐treated mice. The analysis revealed that MgC^@PS^ treatment downregulated pro‐inflammatory genes involved in the NLRP3 pathway, NFκB pathway, TNF signaling pathway, necroptosis pathway, and others (Figure 2J,K). Meanwhile, genes associated with antioxidant and anti‐inflammatory pathways, such as the FoxO pathway, autophagy pathway, and phagocytosis pathway, showed a trend of up‐regulation. Further investigation of genes related to the NLRP3 signaling pathway, including *Il1b*, *Casp1*, *Pycard*, and *Nfkb1*, indicated higher expression in the Mφ_3 and Mφ_4 subclusters, underscoring the anti‐inflammatory effect of MgC^@PS^ (Figure 2L). Recent studies highlighted that natural killer (NK) cell is tightly correlated with liver‐associated macrophages and liver diseases.^[^ ^57^ ^]^ Thus, we analyzed NK and T cell populations for differentially expressed genes (DEGs). Among NK cells, six subpopulations were identified, with NK_1 and NK_2 subpopulations being predominant in MgC^@PS^ treated mice. A transcriptome trajectory heatmap demonstrated that MgC^@PS^ treatment shifted NK cells from a pro‐inflammatory to an anti‐inflammatory phenotype (Figure S4D, Supporting Information). Anti‐inflammatory markers were highly expressed in NK_1 and NK_2 subpopulations, while pro‐inflammatory markers were localized in NK_3 and NK_5 subpopulations (Figures S4E–G, Supporting Information).

### 2.3 MgC^@PS^ Induces Macrophage Immunological Tolerance, Protects Against Pyroptosis, and Disrupts Crosstalk Between Kupffer Cells and HSCs

**Figure 3:**
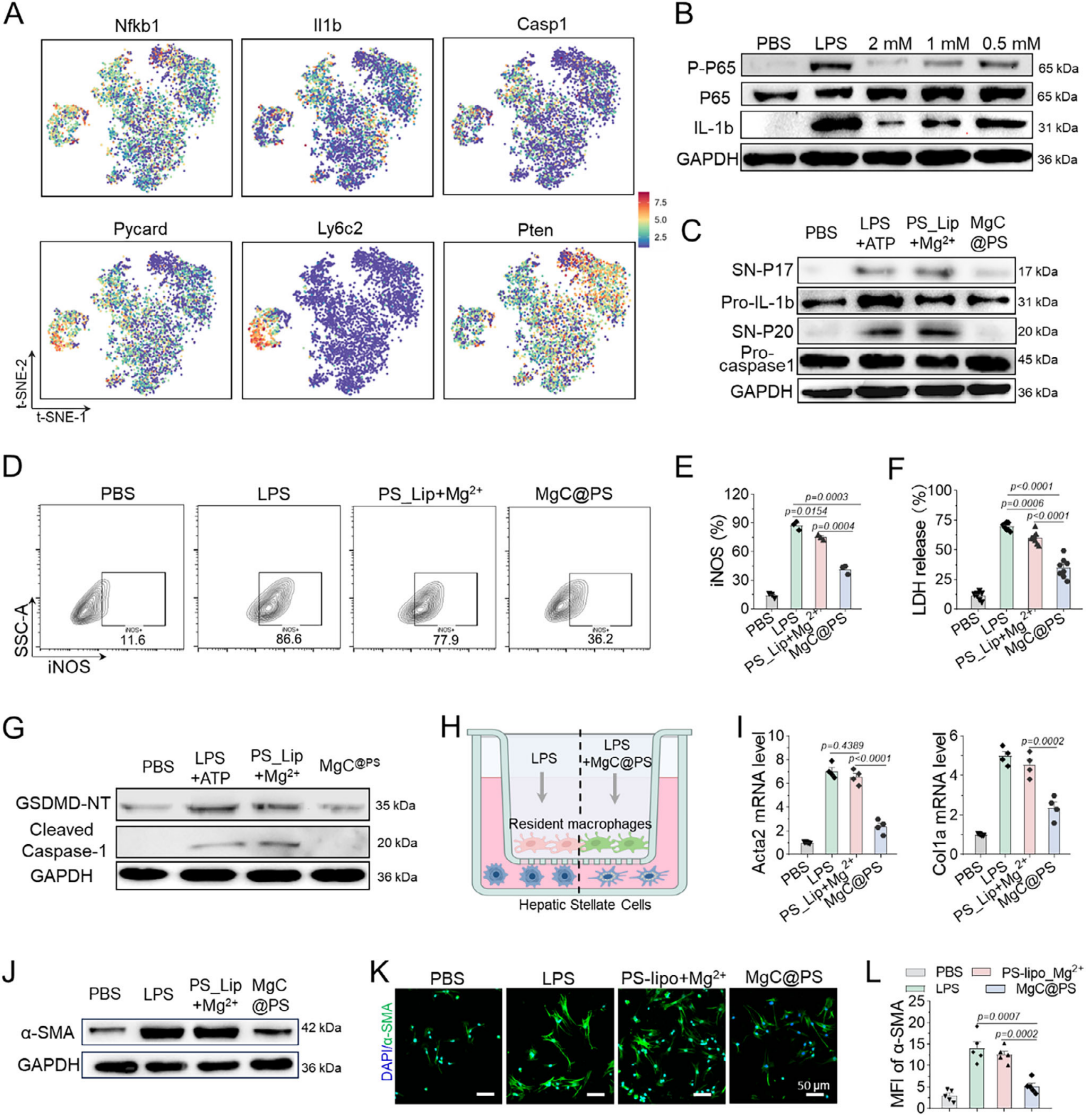
MgC^@PS^ modulates macrophage function, suppresses pyroptosis, and mitigates macrophage‐HSC crosstalk in CCl_4_‐induced inflammation and fibrosis. A) t‐SNE plots illustrating the relative distribution of genes involved in the NOD‐like receptor signaling pathway. B) Western blot analysis showing the phosphorylation of P65 and IL‐1β protein levels in bone marrow‐derived macrophages treated with LPS, with or without MgC^@PS^. C). Western blot analysis of cleaved IL‐1β (P20) and cleaved caspase‐1 (P20) levels in supernatant of THP‐1 cells following LPS (100 ng mL^−1^, 4 h) priming and ATP (5 mm, 1 h) stimulation. MgC^@PS^ was added after 2 h of LPS treatment. D) Flow cytometry analysis of iNOS expression in BMDMs treated with LPS, with or without MgC^@PS^. E) Quantitative analysis of iNOS expression ratios corresponding to panel F (*n* = 3). F) LDH release assay in THP‐1 cells treated with MgC^@PS^, assessing pyroptosis induced by LPS (100 ng mL^−1^, 4 h) + ATP (5 mm, 1 h) (*n* = 8), G) Western blot analysis showing the phosphorylation of GSDMD‐NT and cleaved caspase 1 (P20) levels in Kupffer cells treated with LPS+ATP, with or without MgC^@PS^. H) Schematic diagram of the coculture experiment design (elements generated via BioRender). Primary liver macrophages were seeded in the upper chamber, and HSCs were seeded in the lower chamber. After incubation for 36 h, HSCs were analyzed by various methods. I) Normalized mRNA expression levels of *Acta2* and *Col1a* in primary mouse HSCs cocultured with liver‐resident macrophages, with or without LPS and MgC^@PS^ treatment (*n* = 4). J) Western blot analysis of α‐SMA protein levels in HSCs after coculture with liver macrophages subjected to different treatments. K,L) Representative immunofluorescence staining of α‐SMA in primary mouse HSCs under various treatments, with corresponding statistical analysis of α‐SMA MFI shown in panel L (*n* = 5). Data are presented as mean ± S.D.

Building on the scRNA‐seq results and KEGG analysis, we further examined NLRP3‐related and other inflammatory genes, which exhibited higher expression in the Mφ_3 and Mφ_4 populations (**Figure** **3**A). In vitro analysis of protein expression after LPS stimulation with MgC^@PS^ treatment revealed that MgC^@PS^ effectively inhibited NFκB phosphorylation and IL‐1β production in a dose‐dependent manner (Figure 3B). To evaluate the secretion of pro‐inflammatory cytokines, we treated LPS‐primed and ATP‐stimulated THP‐1 cells with MgC^@PS^. These cells exhibited high levels of IL‐1β, TNF‐α, and IL‐6, while MgC^@PS^ co‐incubation significantly reduced expression of these pro‐inflammatory cytokines (Figure S5, Supporting Information). Western blot (WB) analysis of cleaved IL‐1β (P17) and cleaved caspase‐1 (P20) in the supernatant, indicative of NLRP3 inflammasome activation.^[^ ^58^, ^59^ ^]^ showed marked inhibition of P17 and P20 secretion with MgC^@PS^ treatment, suggesting that Mg^2^⁺ enrichment suppresses inflammasome formation (Figure 3C). To further assess macrophage polarization, we measured iNOS expression via flow cytometry. LPS treatment induced a pro‐inflammatory polarization state, with ≈86.6% inducible nitric oxide synthase (iNOS) expression after 6 h. In contrast, MgC^@PS^ treatment reduced ≈57.7% iNOS expression compared to LPS‐treated BMDM cells alone (Figure 3D,E). We also evaluated lactate dehydrogenase (LDH) release as a marker of pyroptosis in THP‐1 cells. MgC^@PS^ treatment significantly mitigated LDH release induced by LPS and ATP, reducing it from ≈71.4% to ≈35.1%, a ≈50% reduction (Figure 3F). We further analyzed anti‐inflammation and anti‐proptosis effect of MgC^@PS^ on Kupffer cells isolated from liver tissues after LPS + ATP treatment. We found that MgC^@PS^ showed treatment inhibited the cleaved caspase 1 formation and cleaved N‐terminal gasdermin D (GSDMD‐NT) production, which indicates the efficient performance of MgC^@PS^ on the Kupffer cell population from pyroptosis (Figure 3G). To better mimic the in vivo environment, primary macrophages were isolated from normal mouse livers to evaluate the anti‐inflammatory effects of MgC^@PS^ and its impact on macrophage‐HSC crosstalk. Using a transwell coculture system, liver‐resident macrophages were seeded in the apical compartment of the inserts, and HSCs were seeded in the basolateral chamber (Figure 3H). After 36 h of LPS stimulation, HSCs showed a significant increase in *Acta2* and *Col1a* mRNA levels (encoding alpha‐smooth muscle actin (α‐SMA) and collagen‐I, key markers of fibrosis) (Figure 3I), which indicated the fibrosis progression. Protein expression evaluated by WB analysis confirmed a similar trend that exhibited reduced α‐SMA levels upon MgC^@PS^ treatment (Figure 3J–L).

### 2.4 Enhanced Amelioration of CCl_4_ Induced Liver Injury and Fibrosis by MgC^@PS^

**Figure 4:**
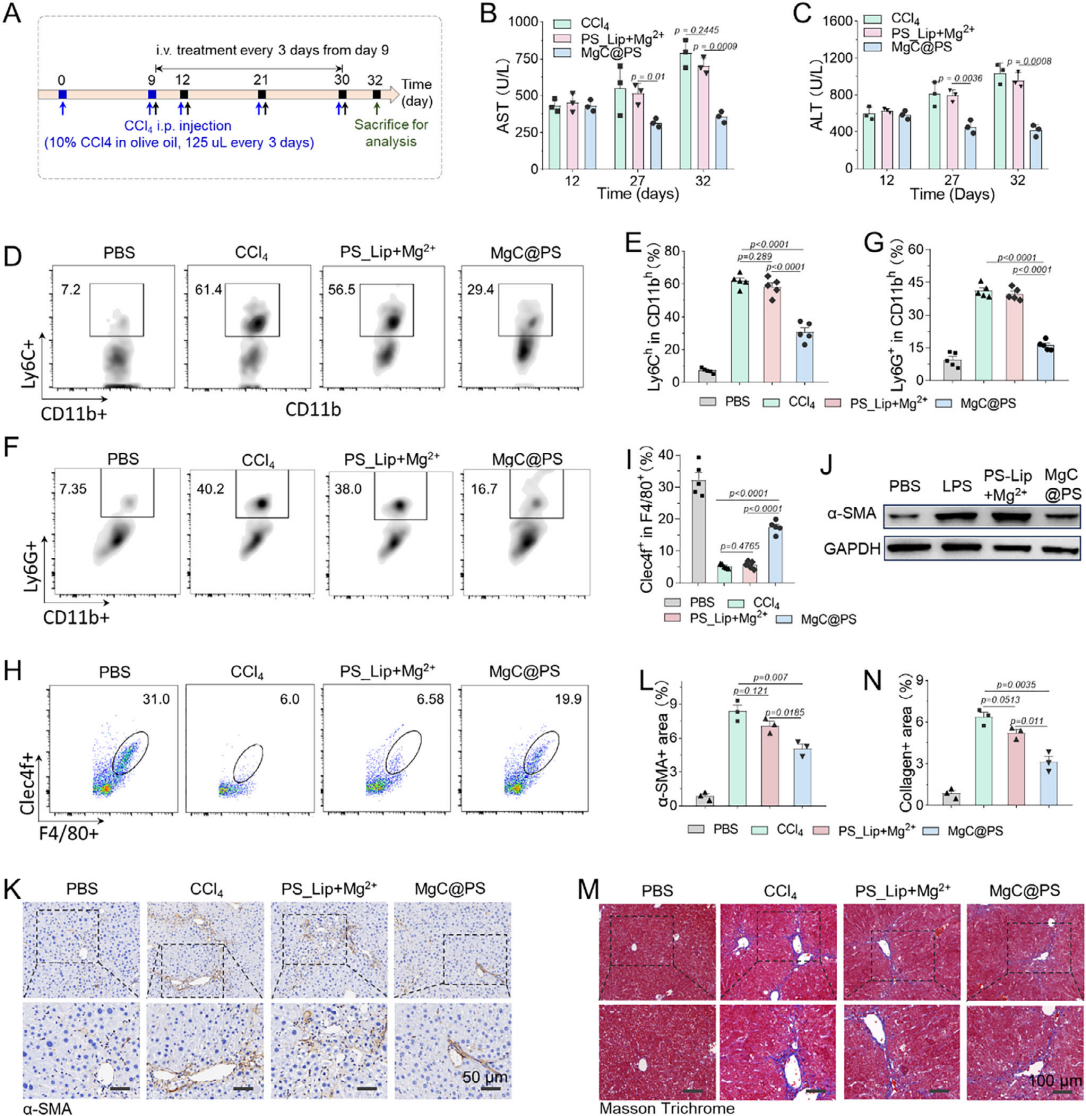
MgC^@PS^ reduces inflammation, preserves tolerogenic Kupffer cells, and protects against fibrosis in CCl_4_‐induced liver injury. A) Experimental time schedule illustrating the induction of liver injury by CCl_4_ and subsequent treatment with MgC^@PS^. B,C) Serum alanine transaminase (ALT) and aspartate aminotransferase (AST) levels during treatment, indicating changes in hepatocellular integrity (*n* = 3). D,E) Flow cytometry analysis of infiltrating Ly6C⁺ inflammatory monocytes and quantification of Ly6C^high^ cell ratios in liver tissues (*n* = 5). F,G) Flow cytometry analysis of infiltrating Ly6G⁺ populations, with or without MgC^@PS^ treatment, and quantification of Ly6G⁺ cell ratios (*n* = 5). H,I) Flow cytometry evaluation of Clec4f⁺ Kupffer cells and calculation of their proportions in liver tissues following treatment (*n* = 5). J) Western blot analysis of α‐SMA expression in liver tissues, reflecting the activation state of hepatic stellate cells (HSCs). K,L) Representative immunohistochemical staining images of α‐SMA⁺ HSCs and their quantification, indicating changes in HSC activation (*n* = 3). M,N) Representative Masson's trichrome staining images demonstrating collagen deposition in liver tissues and quantification of collagen‐positive areas, illustrating fibrosis progression and its attenuation by MgC^@PS^ treatment (*n* = 3). Data are presented as mean ± S.D.

To evaluate the anti‐inflammatory efficacy of macrophage‐targeted Mg^2^⁺ enrichment by MgC^@PS^, we established a CCl_4_‐induced inflammation and fibrosis model (**Figure** **4**A). During CCl_4_ administration, serum alanine transaminase (ALT) and aspartate aminotransferase (AST) levels significantly increased, reflecting hepatocyte injury caused by CCl_4_ and elevated inflammatory cytokines (Figure 4B,C). After three weeks of MgC^@PS^ treatment, these liver damage‐associated enzymes decreased, likely due to Mg^2^⁺‐mediated suppression of pro‐inflammatory macrophage activation. Kupffer cells, a self‐renewing, liver‐resident macrophage population derived from erythromyeloid progenitors, play a pivotal role in maintaining liver immune and metabolic homeostasis.^[^ ^60^ ^]^ To examine the impact of Mg^2^⁺ enrichment on liver‐resident macrophages, we collected and digested liver tissues to isolate non‐parenchymal cells following MgC^@PS^ treatment for flow cytometry analysis (Figure S6A, Supporting Information). Inflammatory cytokines secreted by Kupffer cells recruit circulating monocytes and exacerbate liver inflammation. Prolonged CCl_4_ exposure induced ≈61.4% of infiltrating monocytes to differentiate into a pro‐inflammatory Ly6C^high^ phenotype. In contrast, MgC^@PS^ treatment alleviated the inflammatory microenvironment, reducing monocyte recruitment and differentiation to ≈29.6% (Figure 4D,E). By comparison, PS_Lip+Mg^2^⁺ treatment displayed limited anti‐inflammatory effects, likely due to insufficient Mg^2^⁺ enrichment in macrophages. A similar trend was observed for the Ly6G⁺ population (Figure 4F,G). CCl_4_‐induced hepatocyte damage triggered robust inflammatory responses in Kupffer cells, leading to an ≈88% loss of their tolerogenic Clec4f⁺ phenotype (Figure 4H,I), which exhibited a concordant trend with scRNA seq results (Figure S6B, Supporting Information). This reduction may stem from pyroptosis driven by oxidative stress, high ATP levels, and pathogen infiltration due to compromised liver barriers. Remarkably, MgC^@PS^ administration preserved ≈64% of the Clec4f⁺ macrophage population. Sustained macrophage‐driven inflammation further activated HSCs, increasing their expression of scar‐associated proteins (e.g., α‐SMA, collagen). Western blot and immunohistochemical analyses of liver tissues revealed a substantial rise in α‐SMA expression following CCl_4_ treatment. However, MgC^@PS^ administration mitigated α‐SMA production, likely by disrupting the pro‐fibrotic crosstalk between hepatic macrophages and HSCs (Figure 4J–L). Additional immunohistochemical staining and Masson trichrome assays confirmed that chronic CCl_4_ exposure induced severe liver fibrosis and abundant collagen deposition, both of which were significantly alleviated by the targeted enrichment of Mg^2^⁺ into macrophages (Figure 4M,N).

### 2.5 Cord Blood Stem Cell Vesicle Derived Hybrid Nano Mg Nourisher (MgC^@PS_SCV^) Effectively Protects Intestinal Barrier and Suppressed Gut‐Liver Co‐Existing Inflammation

Bidirectional communication of the gut‐liver axis can cause liver and biliary complications.^[^ ^44^, ^61^, ^62^, ^63^, ^64^, ^65^ ^]^ Thus, we further investigated the performance of MgC^@PS^ in treating intestinal inflammation in the context of liver injury. The dual‐disease model (liver injury and ulcerative colitis) was established by combining treatment of i.p. administration of CCl_4_ and oral feeding of DSS in drinking water (Figure S7A, Supporting Information). In DSS‐induced ulcerative colitis, goblet cells and epithelial cells are damaged, causing a disrupted intestinal barrier that is constructed by a mucus layer and tight junction, which further allows pathogen and endotoxin permeation, followed by abundant inflammatory macrophage infiltration. Consistent with its effective anti‐inflammatory function in addressing liver injury, MgC^@PS^ demonstrated notable anti‐inflammatory performance as evidenced by the inhibition of pro‐inflammatory macrophage infiltration as well as cytokine production in colon tissues (Figure S7B–D, Supporting Information). However, the mice showed body weight loss and noticeable colon length reduction (Figure S7E–G, Supporting Information). Meanwhile, colon tissues exhibited compromised intestinal structure integrity caused by DSS (Figure S6H; Figure S7I, Supporting Information). We hypothesize that while MgC^@PS^ can address the inflammatory phenotype of macrophages in the intestine, it does not enhance tissue regeneration following DSS‐induced epithelial damage. To further investigate MgC^@PS^ impact on macrophage‐mediated epithelial integrity restoration, we conducted in vitro studies on tight junction‐related protein expression after DSS treatment, with or without MgC^@PS^. Tight junction acts as a gatekeeper that is crucial to block pathogens and toxins, maintaining the epithelial integrity and intestinal homeostasis.^[^ ^66^ ^]^ CaCO‐2 with DSS treatment was cocultured with THP‐1 cells and MgC^@PS^ for 24 h, and was applied to evaluate the tight junction‐related protein, Occludin. CaCO‐2 cells exhibited reduced expression of these proteins, suggesting that macrophage‐mediated protection of epithelial integrity was limited (Figure S8A, Supporting Information). Transwell assay for evaluating CaCO‐2 cell monolayer permeability showed a similar trend, DSS treatment caused significantly high permeation (3.5‐fold higher than normal cell monolayer) of FITC‐dextran (4 kDa) from apical to basolateral chamber even in the presence of MgC^@PS^ (Figure S8B, Supporting Information), indicating the loss of tight junction. These findings suggest that MgC^@PS^ exhibited limited potential to induce macrophage‐mediated epithelial monolayer recovery.

**Figure 5:**
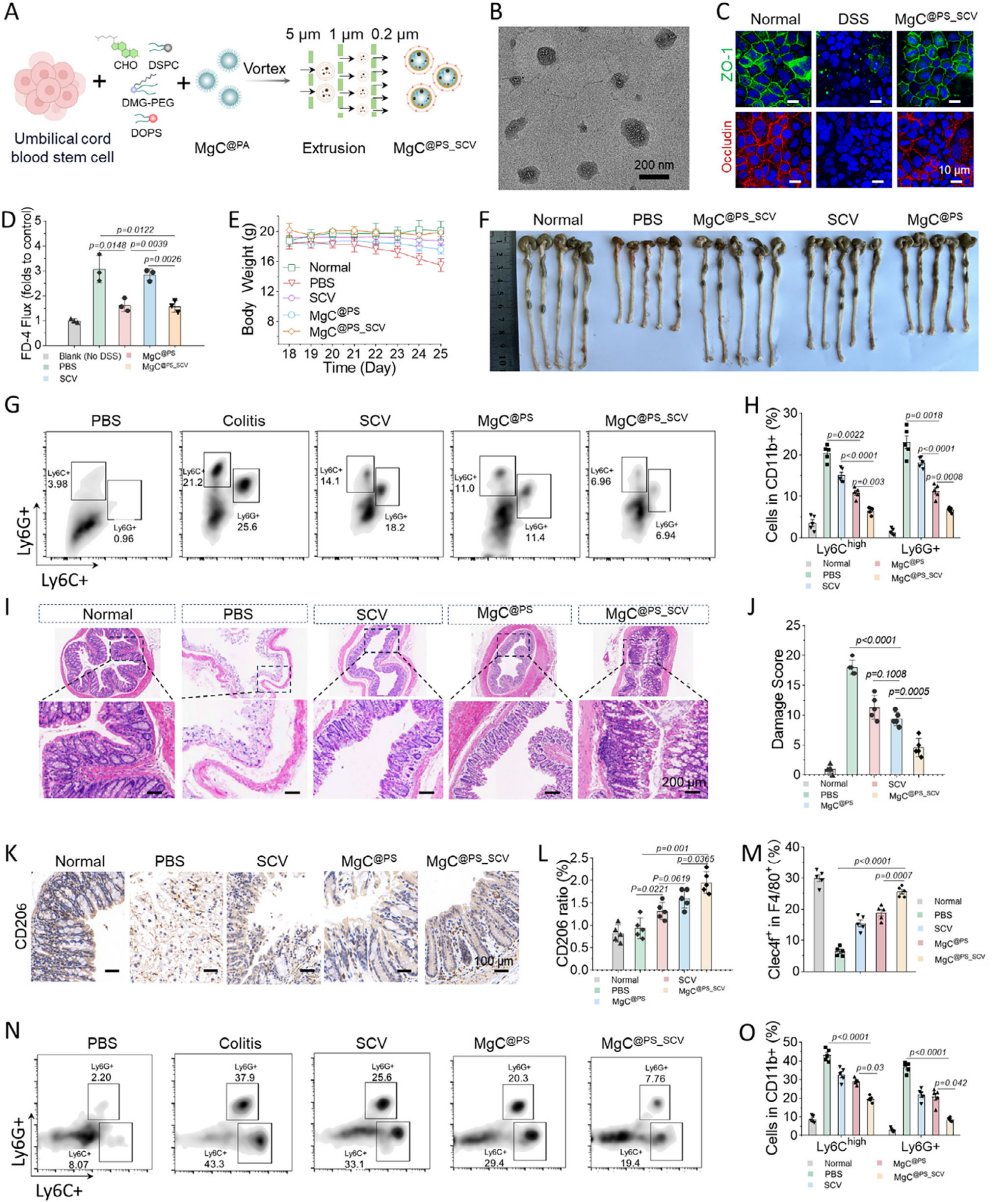
MgC^@PS_SCV^ effectively protects the intestinal barrier and suppresses gut‐liver co‐existing inflammation. A) Scheme showing procedures of MgC^@PS_SCV^ preparation. B) Typical TEM images of MgC^@PS_SCV^. C) CLSM images of tight junction protein expression (ZO‐1 and Occludin) after DSS and MgC^@PS_SCV^ treatment. D) Fluorescence intensity of FITC in basolateral medium indicating the perfused FITC‐dextran from the apical chamber that crosses the CaCO‐2 monolayer after treatment of DSS+MgC^@PS_SCV^ (*n* = 3). E) Development of body weights during UC treatment (from day 18—day 24) (*n* = 5). F) Photographic images of the colon tissues of the normal mice and treated mice. G,H) Flow cytometry analysis of infiltrating inflammatory monocytes and calculation of cell ratios in the lamina propria of colon tissues after MgC^@PS_SCV^ treatment (*n* = 5). I,J) Typical hematoxylin and eosin (H&E) stain images and damage scores of colon tissues after treatments (*n* = 5). K,L) Typical images of immunohistochemical (IHC) staining for CD206+ expression visualization and statistical calculation of CD206+ cell ratio in colon tissues (*n* = 5). M) Flow cytometry evaluation of Clec4f+ Kupffer cells and calculation of their proportions in liver tissues following treatment (*n* = 5). N,O) Flow cytometry analysis of infiltrating inflammatory monocytes and calculation of cell ratios in liver tissues after MgC^@PS_SCV^ treatment. Data are presented as mean ± S.D (*n* = 5).

Therefore, further optimization of our Mg ion‐based nourisher is essential to accelerate the restoration of the intestinal barrier, which plays a critical role in preventing toxin and pathogen permeation into systemic circulation and reducing liver burden.^[^ ^67^ ^]^ Recent studies have reported that stem cell‐derived vesicles (SCVs) exhibited potential to educate macrophages for enhancing tissue regeneration.^[^ ^68^, ^69^, ^70^ ^]^ Moreover, their low immunogenicity makes them excellent therapeutic candidates for tissue damage repair.^[^ ^71^ ^]^ Building on the anti‐inflammatory properties of MgC^@PS^ and the tissue‐regenerative potential of stem cells, we developed a nanosized hybrid SCV‐based Mg nourisher (MgC^@PS_SCV^) for the treatment of liver‐gut inflammation that was achieved by extruding SCVs with an MgC core in the presence of DOPS (**Figure** **5**A). DLS measurements revealed that MgC^@PS_SCV^ had a mean diameter of 155.6 nm, and TEM images showed a uniform structure (Figure 5B; Figure S8C, Supporting Information). MgC^@PS_SCV^ showed effective macrophage‐targeted potential in BMDM and Kupffer, while almost no uptake in HSC and hepatocyte (Figure 5D). Meanwhile, colon tissues in UC mice showed time‐dependent accumulation increase (Figure S8E,F, Supporting Information). We further investigated CaCO‐2 monolayer integrity and the tight junction protein expression after MgC^@PS_SCV^ treatment in the presence of THP‐1 cells. MgC^@PS_SCV^ exhibited effective enhancement of the macrophage‐mediated protection on tight junction integrity and blocked FITC‐dextran perfusion (Figure 5C,D; Figure S8G,H, Supporting Information). In the liver‐gut coexisting disease model, mice were administered MgC^@PS_SCV^ every 3 days (for a total of six injections) during CCl_4_ treatment, followed by daily MgC^@PS_SCV^ injection during 3% DSS feeding (Figure S8I, Supporting Information). Under the DSS challenge, mice in the PBS group showed persistent body weight loss, progressive diarrhea, and rectal bleeding (Figure 5E). In contrast, MgC^@PS_SCV^ treatment‐maintained body weight and mitigated these clinical signs, reflecting its anti‐inflammatory efficacy. After treatment, the colon tissues were resected and photographed, revealing marked colon shortening and loose stools in DSS‐treated mice (Figure 5F; Figure S8J, Supporting Information). Flow cytometry analysis of lamina propria (LP) cells indicated a pronounced increase in CD11b^+^ Ly6C^high^ and CD11b^+^ Ly6G^+^ inflammatory monocytes, likely stemming from barrier dysfunction and dysbiosis (Figure 5G,H; Figure S8K, Supporting Information). MgC^@PS_SCV^ treatment alleviated this exacerbated inflammation by inhibiting monocyte differentiation to Ly6C^high^ inflammatory phenotypes (67% inhibition compared to the PBS group). Histological examination via Hematoxylin and Eosin (H&E) staining showed extensive goblet cell loss, erosion, hyperplasia, and ulceration in the mucosa, submucosa, and LP layers of DSS‐treated mice (Figure 5I,J). Immunostaining for the anti‐inflammatory marker CD206 revealed that both MgC^@PS^ and MgC^@PS_SCV^ treatments enhanced CD206 expression (Figure 5K), suggesting that Mg^2+^ enrichment can shift macrophage polarization and ameliorate colitis. Quantification showed a 2.2‐fold increase in CD206^+^ cells under MgC^@PS_SCV^ treatment (Figure 5L). Flow cytometric analysis of liver tissues demonstrated that MgC^@PS_SCV^ provided stronger anti‐inflammatory protection, notably preserving Kupffer cells from pyroptosis (Figure 5M; Figure S8L,M, Supporting Information). Concurrent CCl_4_ and DSS treatment led to pronounced infiltration of Ly6G^+^ and Ly6C^high^ monocytes into liver tissues. Although MgC^@PS^ and SCV treatments each reduced inflammatory monocyte infiltration moderately (Figure 5N,O), MgC^@PS_SCV^ treatment exhibited a substantial inhibition of infiltrating monocytes. Overall, these findings highlight the potential of MgC^@PS_SCV^ as a robust therapeutic platform for reducing hepatic inflammation and intestinal damage in the gut‐liver dual‐disease model.

## 3 Discussion

Chronic inflammatory diseases, and inflammatory bowel disease (IBD), impose a substantial global health burden.^[^ ^40^, ^41^, ^42^, ^43^, ^44^, ^45^, ^46^, ^47^ ^]^ Macrophages are at the central stage of diverse pathological processes driving the prognosis of chronic inflammatory diseases. It induces pyroptosis through NLRP3 inflammasome activation, causes cytokine storms, and promotes fibrotic progression along the gut‐liver axis.^[^ ^27^, ^28^, ^29^, ^30^, ^31^, ^32^, ^33^, ^34^ ^]^ Mg^2^⁺ emerges as a promising inflammation modulator, was found to be inversely correlated with disease severity.^[^ ^21^ ^]^ Current Mg^2^‐based treatments have limitations in poor intracellular bioavailability, off‐target effects, and hypermagnesemia risks, constraining their therapeutic translation. Our work engineered a phosphatidylserine‐coated magnesium nano‐nourisher (MgC^@PS^) that leverages efferocytosis for selective iMg^2^⁺ enrichment in tissue‐resident macrophages, yielding potent NLRP3 inhibition and phenotypic reprogramming. Single‐cell RNA sequencing analysis revealed a shift from pro‐inflammatory subclusters (Mφ_3/Mφ_4, enriched in Il1b and Casp1) to anti‐inflammatory Mφ_2 (upregulated Mrc1, Cd163, Hmox1), with FoxO/autophagy pathway enrichment bolstering antioxidant resilience. MgC^@PS^ dose‐dependently quenched NF‐κB phosphorylation, cleaved caspase‐1 (p20), IL‐1β (p17), LDH release, and iNOS expression in LPS/ATP‐stimulated THP‐1 cells, preserving tolerogenic states. In the CCl_4_ liver injury model, MgC^@PS^ effectively preserved Clec4f^+^ Kupffer cell population and halved Ly6C^high^ monocyte infiltration, disrupting fibrotic crosstalk without affecting hepatocytes.

Our results suggest two non‐exclusive mechanisms underlying the anti‐inflammatory role of MgC^@PS^. On one hand, MgC^@PS^ blunts NLRP3 priming and activation through iMg^2^‐dependent intracellular signaling regulation. Meanwhile, MgC^@PS^ is able to reprogram macrophages to a pro‐resolution phenotype that reinforces tissue homeostasis. This represents a clear advancement over current inflammasome‐targeted strategies, including IL‐1 pathway blockers and small‐molecule NLRP3 inhibitors, which are known to have narrow therapeutic windows and adverse systemic impact. The novelty of MgC^@PS^ lies in its ability to localize an endogenous immunonutrient to macrophages, significantly widening the safety margin while engaging tissue repair programs. Its safety profile was evidenced by the absence of hepatocyte injury and preservation of resident Kupffer cells in our models.

Due to the bidirectional communication of the gut‐liver axis, liver inflammatory diseases often coexist with intestinal inflammation, as liver injury can induce a proinflammatory immune profile that disrupts the intestinal barrier, with 5–10% of inflammatory bowel disease patients developing liver and biliary complications.^[^ ^48^, ^49^, ^61^, ^62^, ^63^, ^64^, ^65^ ^]^ Challenges persist in achieving dual anti‐inflammatory and regenerative efficacy, particularly in interconnected gut‐liver pathologies where barrier dysfunction perpetuates endotoxin translocation and NLRP3 priming. Extending to the gut‐liver axis, MgC^@PS^ curbed colonic CD11b⁺ Ly6C^high^ accumulation in DSS‐colitis but showed limited epithelial repair (persistent ZO‐1/Occludin downregulation and 3.5‐fold FITC‐dextran permeability). Thus, we developed a hybrid magnesium nano‐nourisher with cord blood stem cell vesicles (MgC^@PS_SCV^) that retained macrophage tropism while aiming for amplified regeneration. MgC^@PS_SCV^ was able to restore CaCO‐2 tight junctions and block ≈70% dextran leakage in co‐cultures. In the CCl_4_/DSS caused liver‐gut dual injury disease model, MgC^@PS_SCV^ alleviated ulceration, boosting CD206⁺ macrophage population and significantly suppressing Ly6C^high^ infiltration, maintaining barrier integrity and immune homeostasis.

Overall, MgC^@PS^ and its SCV hybrid pioneer nutritional metal‐based anti‐inflammatory treatment by suppressing NLRP3 activation, offering a powerful platform to tame macrophage‐driven inflammation and foster tissue recovery in gut‐liver axis‐related diseases.

## 4 Statistical Analysis

Statistical analysis was performed using Prism 8.0 (GraphPad, USA). An unpaired two‐sided Student's *t*‐test was used for comparison between two groups. For data with multiple groups, the statistically significant differences were assessed using one‐way analysis of variance (ANOVA) or two‐way ANOVA with Tukey's multiple comparisons test.

## Conflict of Interest

The authors declare no conflict of interest.

## Supporting information



Supporting Information

## Data Availability

Data supporting this study are provided in the supplementary material and available from the authors upon request.
