# Supplementary material for: Macrophage‐Targeted Magnesium Ion‐Nourisher for NLRP3 Inflammasome Inhibition to Enhance Liver Inflammatory Disease Treatment
Source: Adv Sci (Weinh). 2025 Nov 5;13(5):e13798. doi: 10.1002/advs.202513798 (PMC12850138; doi:10.1002/advs.202513798)
Supplement: Supplementary file 1 — Supporting Information [file ADVS-13-e13798-s001.docx]

Supporting Information

Macrophage-Targeted Magnesium Ion-Nourisher for NLRP3 Inflammasome Inhibition to Enhance Liver Inflammatory Disease Treatment

Li Wang, Zhuo Yan, Sindhu Yalavarthi, Yusif Abdul-Rashid, Kiki Parker, Kyle Nowlin, Ethan Li, Jordan Mack, Josephine Wei, Hunter Vu, Zhenquan Jia, Jianjun Wei, Jilong Wang^*^ and Kerui Wu^*^

Dr. L. Wang, S. Yalavarthi, Y. Rashid, K. Nowlin, E. Li, J. Mack, H. Vu, J. Wei, Dr. J. Wei, Dr. K. Wu,

Department of Nanoscience, Joint School of Nanoscience and Nanoengineering, University of North Carolina at Greensboro, Greensboro, NC 27401, USA

*E-mail: k_wu@uncg.edu

Z. Yan, Dr. J. Wang,

Joint Centre of Translational Medicine, The First Affiliated Hospital of Wenzhou Medical University, Wenzhou Medical University, Wenzhou, Zhejiang 325000, China

*E-mail: wangjilong@wmu.edu.cn

K. Parker

Department of Nanoengineering, North Carolina Agricultural and Technical State University, Greensboro, NC, 27401, USA

Dr. Z. Jia,

Department of Biology, University of North Carolina at Greensboro, Greensboro, NC 27412, USA

H. Vu

J. Crayton Pruitt Family Department of Biomedical Engineering, University of Florida, Gainesville, FL 32611, USA

Keywords: (inflammation, macrophage, magnesium nano-nourisher, NLRP3 inflammasome, stem cell-derived vesicles)

**Materials.**

Magnesium chloride (MgCl_2_), Na_2_HPO_4_, cyclohexane, igepal-co-520, ethyl alcohol, chloroform, EDTA were purchased from Millipore Sigma (Saint Louis, MO). DSPC (1,2-distearoyl-sn-glycero-3-phosphocholine, 18:0 PC), cholesterol, DMG-PEG_2000_ (1,2-dimyristoyl-rac-glycero-3-methoxypolyethylene glycol-2000), 1,2-dioleoyl-sn-glycero-3-phospho-L-serine (sodium salt) (DOPS) and 1,2-dioleoyl-sn-glycero-3-phosphate (sodium salt) (DOPA) were purchased from Avanti Polar Lipids (Birmingham, AL). Hoechst 33342, and 2-(4-amidinophenyl)-6-indolecarbamidine dihydrochloride (DAPI) were purchased from Millipore Sigma (Saint Louis, MO, USA). HBSS were purchased from Gibco (Grand Island, NY). Water was purified by using a Milli-Q purification system (Millipore, Bedford, MA). Other chemicals or solvents without mention were analytical grade and used as received. Antibodies against cell surface markers for flow cytometry assay were obtained from Biolegend (San Diego, CA). Other chemicals and reagents were of analytical grade and used as received, unless noted otherwise.

**Characterization.**

Magnesium content was determined on an Inductively Coupled Plasma Mass Spectroscopy (ICP-MS, Agilent 7900, Santa Clara, CA). The morphology and element mapping of MgC^@PS^ was analyzed by a transmission electron microscope (TEM, Hitachi HT-7700, Japan) at an accelerating voltage of 120 kV. Particle size and zeta potential measurements were conducted using a zeta potential analyzer with dynamic light-scattering capability on a Malvern Zetasizer Nano ZS90 (Malvern Instruments, UK) with He-Ne laser (633 nm) and 90° collecting optics. Data were analyzed using Malvern Dispersion Technology Software 7.02.

**MgC^@PS_SCV^ preparation and determination of Mg content**.

The DOPS-coated MgC nanoparticles (MgC^@PS^) were synthesized using a water-in-oil reverse microemulsion method. The lipid composition included DSPC, cholesterol, DOPS, and DMG-PEG_2000_. The inner layers of the liposome membrane were formed from DOPA. In brief, 1 mL of MgCl_2_ (2.5 M) was added to 50 mL of mixed oil phase 1 (cyclohexane: Igepal CO-520 = 75:25) in a 250 mL flask, and the solution was stirred at 1000 r.p.m. for 3 minutes to generate a well-dispersed microemulsion phase. For oil phase 2, 300 μL of DOPA lipid (20 mM) were added to 50 mL of the same mixed oil phase. A separate microemulsion containing sodium carbonate was prepared by adding 600 μL of Na_2_CO_3_ (50 mM) to 50 mL of mixed oil phase 2, maintaining a magnesium (Mg) to Carbonate (C) molar ratio of 50:1. The MgC core with a single layer of DOPA was formed by rapidly mixing oil phase 1 and oil phase 2, followed by stirring at 1000 r.p.m. for 15 minutes. To collect the DOPA coated MgC cores, 50 mL of ethanol was added to the mixture, which was then centrifuged at 8,000×g for 15 minutes, followed with ethanol wash. The collected MgC^@PA^ were resuspended in 4 mL of chloroform. To prepare MgC^@PS^, MgC with inner layer lipid that resuspending in chloroform was added with 1mL lipid cocktail (DOPS: DSPC: Cholesterol, DMG-PEG_2000_ = 4:3:2:1, all lipid concentration = 20 mM, for DiD dye labelling, same amount with DMG-PEG_2000_ of DiD was added in this procedures). After dry chloroform with N_2_ flow, the film was hydrated in 5 mL PBS and sonicated for 5 min at room temperature. To determine the Mg content in the nanoparticles, MgC^@PS^ were dissolved in 1M HNO_3_ and determined by ICP-MS. PS-Lip was prepared by same amount of DOPS, Cholesterol, DSPC and DMG-PEG_2000_ with MgC^@PS^. For DLS measurement, the film was hydrated in water instead of PBS. To prepare other metal nanoparticles, replace MgCl_2_ to CaCl_2_, ZnCl_2_, Ce(NO_3_)_3_ and MnCl_2_, respectively. For CaP and MnP preparation, Na_2_CO_3_ was replaced with Na_2_HPO_4_.

To prepare MgC^@PS_SCV^, MgC^@PA^ were hydrated with 5 mL umbilical cord blood stem cells (National Stem Cell Translational Resource Center, Shang Hai, China) derived vesicles (vesicles were extruded from 2.5×10^7^ stem cells using 5 μm Whatman Nuclepore^TM^ Track-etched Membranes in an Avanti Mini Extruder). After vortex for 30s×20 times, mixture was co-extruded using 1 μm and 0.2 μm Nuclepore^TM^ track-etched membranes with 10 passages. The solution should be uniform without solid aggregates before extrusion. Vortex should be repeated if the aggregates is existed.

**Cell culture and animal use**.

RAW264.7, HUVEC, THP-1, 4T1, CaCO-2 cell lines were obtained from American Type Culture Collection (ATCC, VA) and cultured in a humidified atmosphere at 37 °C with 5% CO_2_. The cells were cultured in normal RPMI 1640 culture medium (Gibco, Thermo Fisher, Grand Island, NY) supplemented with 10% fetal bovine serum (FBS, ExCell Bio, China) and 1% penicillin/streptomycin (Gibco, Life Technologies, Grand Island, NY). CaCO-2 cell line was cultured in EMEM medium with non-essential amino acid addition (Gibco, Thermo Fisher, Grand Island, NY) and 20% FBS.

Primary bone-marrow-derived macrophages (BMDM) were isolated by flushing the tibia and femur of 6 to 8-week-old C57BL/6 mice using ice cold PBS containing 1% heated inactivated FBS, followed by culturing in RMPI 1640 medium containing 10% heat inactivated FBS with addition of 50 ng/mL M-CSF for BMDM. Kupffer cells and hepatocytes were isolated according to a previous reported STAR protocol^[1]^. Typically, after anesthetizing the mouse with isoflurane, 10 mL 0.5 mM EDTA (PH = 7.2) in Ca^2+^ Mg^2+^ free HBSS was perfused into Vena Cava using 25 G needle at 5 mL/min using peristaltic pump. Prewarmed collagenase-IV solution (37℃, 0.75 mg/mL) and DNase I (5 units/mL) in RPMI medium and 2% inactivated FBS were perfused from Vena Cava. Liver tissue was dissociated in dish and further digested with 5 mL perfused solution for 10 min. After filter with 60 μm sterilized nylon mesh, hepatocytes were isolated by low-speed centrifuge (50 g) for 3 times. The pellet (hepatocytes) was collected and seeded in collagen precoated coverslips. The suspension was centrifuged and resuspended in 6 mL 20% percoll and overlaid on the 50% percoll. After centrifuging with 800 g for 15 min in a slow brake, Kupffer cells in the interface were collected and washed with PBS and cultured in RPMI 1640 medium with 10% inactivated FBS containing 25 ng/mL M-CSF.

To separate the HSC from the non-parenchymal cells, cells after perfusion and hepatocyte isolation was gently resuspended in 6 mL 52% Percoll (mixture of 2.7 mL cell suspension and 3 mL 100% percoll). 2 mL cell suspension was under layered a two-step Percoll gradient (4 mL of 50% Percoll, 4 mL of 35% Percoll, and 1mL of PBS-1% BSA) and centrifuged at 800 g for 30 minutes at 4 ℃. The 4 layers from top to bottom are 1 mL PBS-BSA, 35% Percoll, 50% percoll, 52% percoll include cells. Three different cell bands were obtained: one at the top of the Percoll gradient enriched in HSC (fraction 1), one at the interface between the two Percoll cushions mainly enriched in liver endothelia cells (fraction 2), and one on the bottom of the tube enriched in kupffers (fraction 3).

Mesenchymal stem cells (UCB-MSCs) were cultured in α-MEM (Thermo Fisher, USA) supplemented with 10% FBS (Gibco, USA) to support growth. To enhance proliferation and maintain stemness, bFGF (5 ng/mL, PEproTech, USA) and PDGF (10 ng/mL, R&D Systems, USA) are added. Medium was changed every 2-3 days to remove non-adherent cells.

All C57/BL6 mice were purchased from Beijing Vital River Laboratory Animal Technology Co., Ltd. All animal procedures were performed in accordance with the guidelines for the Care and Use of the Experimental Animal Ethics Committee of Wenzhou Medical University (wydw2024-0417).

**Coculture of liver resident macrophages and HSCs in transwell**

After perfusion and cell separation, HSCs were seeded in the transwell basolateral chamber with density of 5×10^4^ cells (for fluorescence staining) and 2×10^5^ cells (for qrt-PCR and Western blot) per well (For fluorescence staining of α-SMA, cells were seeded on the round coverslips). Liver resident macrophages were seeded in the transwell apical inserts (0.4 μm polyester membrane; 3470; Costar). The liver resident macrophages were cultured in RPMI 1640 medium. After 30 min preincubation with MgC^@PS^ and 100 ng/mL LPS (LPS from E. coli O111:B4, Invivogen) for 12 h, liver resident macrophages were placed into HSC well for further 36 h co-incubation. The HSCs were harvested to perform further analysis (Immunofluorescence staining, qrt-PCR and Western Blot). For fluorescence staining, the cells were washed, fixed with 4% PFA and stained with α-SMA antibody (1:200, 14-9760-82, Invitrogen) overnight at 4 ℃. After PBS wash for 3 times, cells were incubated with FITC conjugated goat anti-mouse IgG (H+L) secondary antibody (1:3000, 31569, Invitrogen) for 1 h at room temperature. After nuclei staining with DAPI, cells were mounted for confocal laser scanning microscopy (CLSM) observation.

**Cell internalization of MgC^@PS^**

To evaluate efferocytosis of MgC^@PS^, DiD labelled MgC^@PS^ was prepared for fluorescence observation. After seeding cells on seeded on 24-well plates with a density of 5 × 10^4^ cells per well. The medium for BMDM and Kupffer cells seeding contained 20 ng/mL M-CSF. After 12 h, cells were incubated with DiD labelling MgC^@PS^ for 45 min and cells were washed, fixed and stained with phalloidin and DAPI for confocal laser scanning microscopy (CLSM). For HL60 cells, ATRA (0.5μM) was used to induce differentiation for 48 h before MgC^@PS^ incubation.

**Mg^2+^ level fluctuation observation**

To visualize Mg^2+^ level change, 5 × 10^4^ cells were seeded in 35 mm dish with glass bottom (MatTek, MA) overnight. After 30 min incubation with Magnesium Green^TM^ (Thermo Fisher, M3735), AM in Ca^2+^, Mg^2+^ free HBSS and 10 min with Hoechst 33342 (MP Biomedicals, MFCD00012679), cells were washed with HBSS for 3 times to remove free Magnesium Green probe and Hoechst 33342. Cells were further treated with or without LPS (100 μg/mL, InvivoGen, E. coli O111:B4) and MgC^@PS^ and observed by CLSM in real time. Fluorescence images were recorded at scheduled time points.

**Inflammatory stimulation experiment**

THP-1 cells were seeded in 6-well plates at density of 10^6^ per well with 50 ng/mL PMA (Phorbol 12-myristate 13-acetate, HY-18739, MCE) for 36 h. For mere LPS stimulation without ATP treatment, cells were incubated with 100 ng/mL LPS stimulation and MgC^@PS^ (1 mM) or PS_Lip+Mg^2+^ (1 mM) for 6 h. Cells were collected for further analysis. For LPS prime and ATP treatment, all groups of THP-1 cell were primed with 100 ng/mL LPS for 4 h and all formulation treatments were applied at 2 h. After 4 h incubation, 5 mM ATP was added to induce NLRP3 inflammasome formation for 1 h incubation. Cell supernatant was collected for further analysis. MgC^@PS^ were added in 3 h after LPS stimulation. For ELISA assay, THP-1 cells were treated with LPS (100 ng/mL) + MgC^@PS^ for 5 h and 5 mM ATP for 1 h. Cell supernatant were collected for analysis.

**ELISA analysis and lactate dehydrogenase (LDH) assay**

For ELISA assay, cell supernatants were collected and additional centrifugation (8000 g, 5 min) was performed to eliminate any cell debris. The levels of inflammatory cytokines (IL-1β, IL-6 and TNF) were measured according to the manufacturer’s instruction (PeproTech, NJ). For colon tissue cytokine evaluation, colon segment in 50 mM phosphate buffer (pH 6.0) was homogenized (1:10 w/v) at 4 °C. Each sample was centrifuged for 2 × 3 min at 10,000 g at 4 °C. The protein concentration was quantified by BCA assay and the levels of cytokines (TGF-β, (PeproTech, NJ)) and myeloperoxidase (MPO, Elabscience) in the supernatants were measured according to the manufacturer’s instruction. LDH release in supernatant was evaluated by LDH assay kit (Beyotime, Shanghai, China).

**Intestinal barrier permeability evaluation**

CaCO-2 cells were seeded in a transwell chamber with 4.5-μm pores. After cell reach confluency and form monolayer, cells were cultured for 7 days more to allow differentiation. CaCO-2 cells were cocultured with 1.5×10^4^ PMA stimulated THP-1 cells for 6 h. After treatment of 1% DSS (40 kDa, Thermo Fisher Scientific) and other formulation for 24 h, he permeability of cell monolayer measurements was performed by evaluating flux of FITC-dextran (4 kDa, Med. Chem. Express) from apical insert to the basolateral medium. FITC-dextran (1 mg/mL) was added to the apical compartment of the insets. After 6 h of incubation, the basolateral medium aliquots were collected for the measurement of fluorescence at 480 nm excitation and 520 nm emission wavelengths.

**Tight junction protein expression**

CaCO-2 cells were seeded on glass coverslips in 24 wells (10^5^ cells per well) and 6 wells (5×10^5^ cells per well) for confluency and 7 days more to allow differentiation. CaCO-2 cells were cocultured with 3×10^4^ (24 well) and 10^5^ (6 well plate) PMA stimulated THP-1 cells for 6 h. After treatment with 1% DSS and other formulation for 24 h, cells were collected for western blot analysis (ZO-1 (1:500), Occludin (1:500), Claudin 5 (1:500) Abclonal Science Inc., Wuhan, China) or stained with primary antibody (ZO-1 (1:100), Occludin (1:100)) and FITC, Cy3 labeled goat anti rabbit IgG for CLSM observation.

**qRT–PCR**

Total RNA was isolated using Trizol (Invitrogen) or the RNeasy Micro kit (Bio-Rad, CA). Complementary DNA was synthesized using the iScript cDNA synthesis kit (Bio-Rad). PCR was performed using the GoTaq SYBR Green qPCR Master Mix (Promega) and gene-specific primers (Supplementary Data Table 1). Relative expression values were normalized to the average of the controls.

**Western blotting**

After treatment, cells were lysed with RIPA and protein was collected by 15000 g centrifuge. Protein was supplemented with protease inhibitors and phosphatase inhibitor cocktail (MP Biomedicals™, 08W00017). Following BCA assay for protein quantification, 25 μg whole lysates and a Dual Color Standards Protein Ladder (Bio-Rad, 1610394) were loaded in lane of 10% acrylamide/Bis Tris gel for Mw>30 kDa and 14% acrylamide/Bis Tris gel for Mw<30 kDa and transferred to MilliporeSigma™ Immobilon™-P PVDF Membrane (Accessories MilliporeSigma™, IPVH00010). Blots were blocked with 5% BSA and incubated with the primary antibodies, P65 and Phosphorylated P65 (1:1000, 4767T, Cell Signaling Technology), anti-Akt1, anti-Phospho Akt1 (1:1000, 9916T, Cell Signaling Technology), anti-Caspase-1 (1:400, Bioss, bs-10442R), anti-IL-1β (3A6) Mouse mAb (1:600, 12242T, Cell Signaling Technology), anti-GAPDH (1:2000, 5174S, Cell Signaling Technology) at 4 °C overnight and subsequently incubated with the corresponding secondary anti-IgG antibodies, (1:8000) for 1 h at room temperature. Blots were analyzed with an iBright™ FL1500 Imaging System (Thermo Fisher Scientific). For cell secreted protein evaluation by western blot, supernatant was collected and centrifuged at 10000 g for 5 min. After discard the cell debris, supernatant was concentrated by 3 kDa ultracentrifuge tube (Amicon® Ultra Centrifugal Filter, 3 kDa MWCO, Millipore).

**In vivo bio-distribution of MgC^@PS^**

After 3% DSS water feeding, mice were subjected to i.v. administration of MgC^@PS^ (20 mg/kg). After scheduled time, mice were sacrificed and the colons and other main organs were excised to image DiD fluorescence by Perkin Elmer IVIS system. The colon tissue or liver tissue was fixed with 4% PFA for 6 h and dehydrated with 30% sucrose for 12 h. After frozen section, colon tissues were further fixed and stained with F4/80-PE (111604, Biolegend) for macrophage labelling. Tissue nuclei were stained with DAPI and mounted for CLSM imaging.

**Flow cytometry analysis for colon tissues**

After treatment, mice were sacrificed, and the colon tissues were removed and placed in cold Ca^2+^- and Mg^2+^-free HBSS. The mesentery was carefully removed, and the colons were opened longitudinally and washed with cold HBSS. The dissected tissues were cut into small pieces and incubated in HBSS containing 1 mM dithiothreitol (Millipore Sigma, MO) and 5 mM EDTA for 30 minutes at 37°C to remove the epithelial layer. After incubation, the intestinal pieces were washed and placed in HBSS containing 3% FBS, 1 mg/ml (200 U/mL) collagenase-III, and 0.01 mg/ml DNase I (Worthington, NJ) for 1 hour at 37°C. The digested tissues were washed, resuspended in 40% Percoll (GE Healthcare, IL), and layered over a 75% Percoll fraction. Percoll gradient separation was performed by centrifugation at 700 g for 20 minutes at room temperature. Mononuclear cells were collected from the interphase, washed, and resuspended in PBS for antibody labelling, after incubating with fluorescent monoclonal antibody cocktail (CD45, CD11b CD11c, Ly6C, Ly6G) according to manufacturer’s instruction for 30 min at 4 °C, cells were washed and resuspended in PBS containing 0.5% BSA, and 2 mM EDTA (maintain isolated condition) for flow cytometry analysis. The injection dose is 10 mg/kg Mg element according to ICP-MS results. Prior to flow cytometry analysis, we pretreated dissociated intestinal cells with the Dead Cell Removal Kit (Miltenyi Biotec, cat. no. 130-090-101).

**Flow cytometry analysis for liver tissues**

After treatment, mice were sacrificed and liver tissues were excised and ground into single cell suspension (1-2 mm^3^). After digestion with digestion enzyme cocktail (collagenase-I (200 U/ml), collagenase-IV (200 U/mL), and DNAse I (100 μg/mL), Worthington, NJ) at 37 °C for 60 min. Cells were collected and washed with PBS, and resuspended in PBS containing 0.5% BSA and 2 mM EDTA. Cells were stained at 4 °C for 30 min by the addition of an antibody cocktail of fluorescence conjugated antibodies (CD45, CD11b, F4/80, Ly6C, Ly6G, Clec4f (Biolegend, CA)). After washed with cold PBS, cells were resuspended in cold PBS containing 0.5% BSA and 2 mM EDTA for flow cytometry analysis. Prior to flow cytometry analysis, we pretreated dissociated intestinal cells with the Dead Cell Removal Kit (Miltenyi Biotec).

**Blood alanine aminotransferase (ALT) and aspartate aminotransferase (AST) determination.**

Mouse blood samples were collected at predetermined time by retro-orbital blood collection and centrifuged at 2000 rpm for 15 min in room temperature (RT). The serum was collected for further analysis by ALT and AST activity assay kit according to the manufacture manual (Elabscience, China).

**Immunofluorescence (IF) and histological staining**

Liver tissues and colon were excised and rinsed in PBS, and fixed in 4% paraformaldehyde (PFA) 4 h at RT. Tissues were dehydrated with 30% sucrose solution for 18 h at 4 °C. Tissues were embedded in O.C.T. (Fisher Scientific, PA). Tissues were sliced and sections were blocked in 1% BSA at room temperature for 0.5 h. Primary antibodies or fluorescent primary antibody were incubated at 4 °C overnight, and fluorescent secondary antibodies were incubated at 25 °C for 2 h. Finally, the tissues were mounted with DAPI/Antifade Solution (Millipore Sigma, MO).

**Immunohistochemistry assays and Masson Trichrome assays**

After treatment, mice were sacrificed and liver tissues were excised, fixed with 4% formaldehyde and dissected into 6 μm thick sections after embedded in paraffin. Collagen in liver tissues was stained with Masson Trichorme staining kit (Solarbio life sciences, Beijing, China) and visualized by light microscopy, and quantified by Color Deconvolution tool of ImageJ. For immunohistochemical staining of α-SMA, tissue sections were first incubated with primary antibody overnight at 4 °C, then incubated with peroxidase-conjugated secondary antibody as manufacture manual, stained with diaminobenzidine, and imaged with light microscopy.

**Single cell mRNA sequencing**

Mice were treated with PBS or MgC^@PS^ as described and then sacrificed after treatment schedule. Livers were excised, cut into 0.5–1.0 mm³ pieces, washed with 1× PBS, and digested with digestion cocktail (Collagenase IV (0.5 mg/mL) and Collagenase I (0.5 mg/mL)). Cell viability was confirmed to exceed 85% using trypan blue exclusion. Single-cell suspensions were counted using the Countess II Automated Cell Counter and subsequently sorted on a BD FACS Aria™ III after co-staining with CD45 and propidium iodide for live immune cell collection. The single-cell suspensions were processed using a 10X Genomics Chromium instrument to collect 10,000 single cells, following the manufacturer’s instructions for the Chromium Single-Cell 3′ kit. Subsequent cDNA amplification and library construction steps were performed as the standard protocol. Libraries were sequenced on an Illumina NovaSeq 6000 system (paired-end multiplexing run, 150 bp) by LC-Bio Technology Co., Ltd (Hangzhou, China), with a minimum sequencing depth of 20,000 reads per cell. Sequencing data were analyzed using Cell Ranger software (v7.0.0, 10× Genomics), which provided gene expression profiles for each cell. The resulting data were processed using Seurat (v4.1.1) in LC scRNA cloud platform (OmicStudio) for dimensionality reduction, clustering, and single-cell RNA sequencing (scRNA-seq) analysis.

**REFERENCE**

[1] F. Andreata, C. Blériot, P. Di Lucia, G. De Simone, V. Fumagalli, X. Ficht, C. G. Beccaria, M. Kuka, F. Ginhoux, M. Iannacone, *STAR Protocols* **2021**, *2*, 100831.


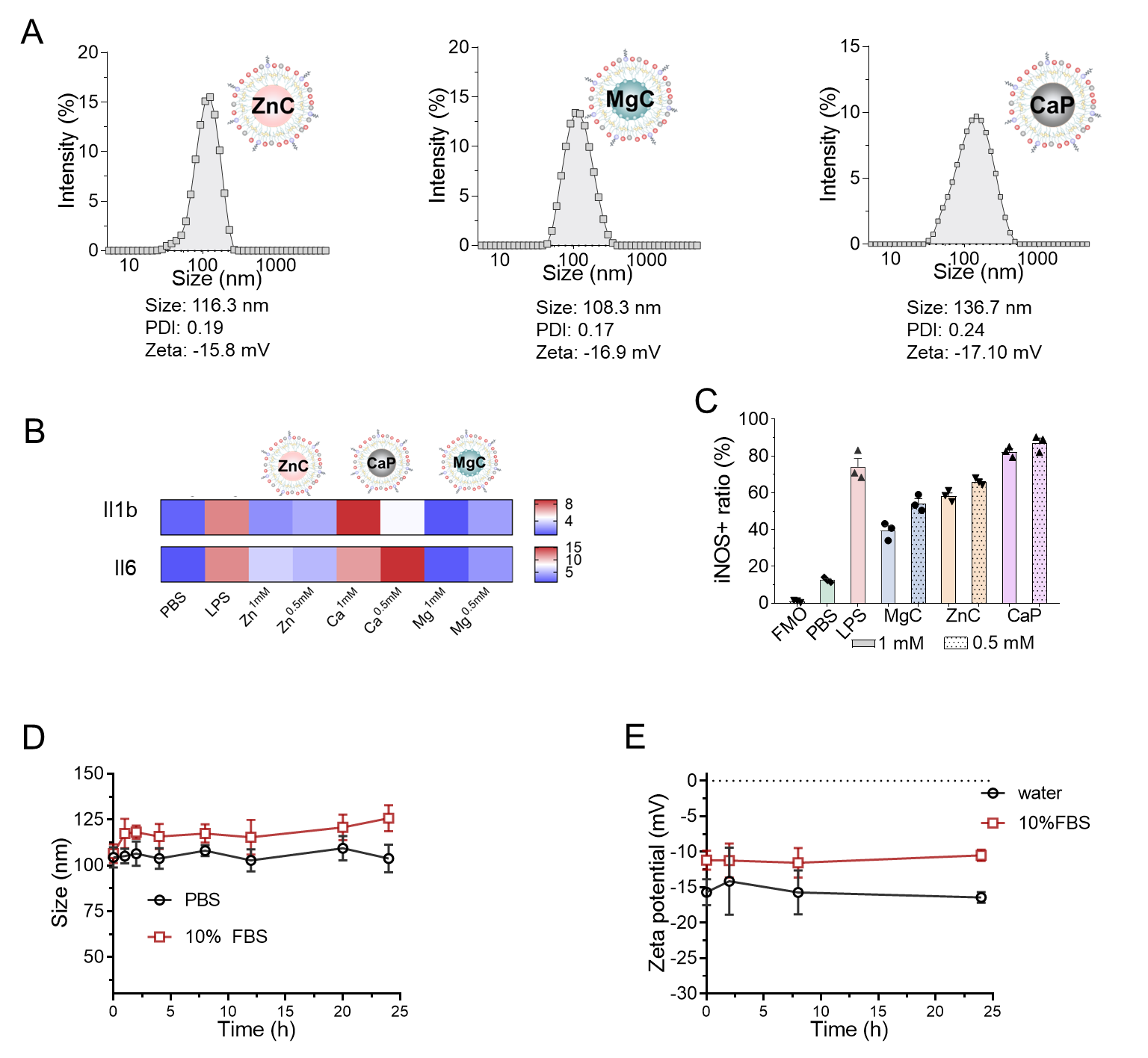


**Figure S1.** (A) DLS measurements of size distribution and zeta potential of three metal nanoparticles. (B) mRNA level of Il1b, Tnf and Il6 after LPS stimulation and various metal nanoparticle treatment in THP-1 cells. (C) Flow cytometry analysis of iNOS expression after LPS stimulation with metal nanoparticle treatment (n = 3). (D) Time dependent size change after incubating with PBS and PBS containing 10% FBS. (E) Time dependent zeta potential change in water and after incubating with PBS containing 10% FBS.


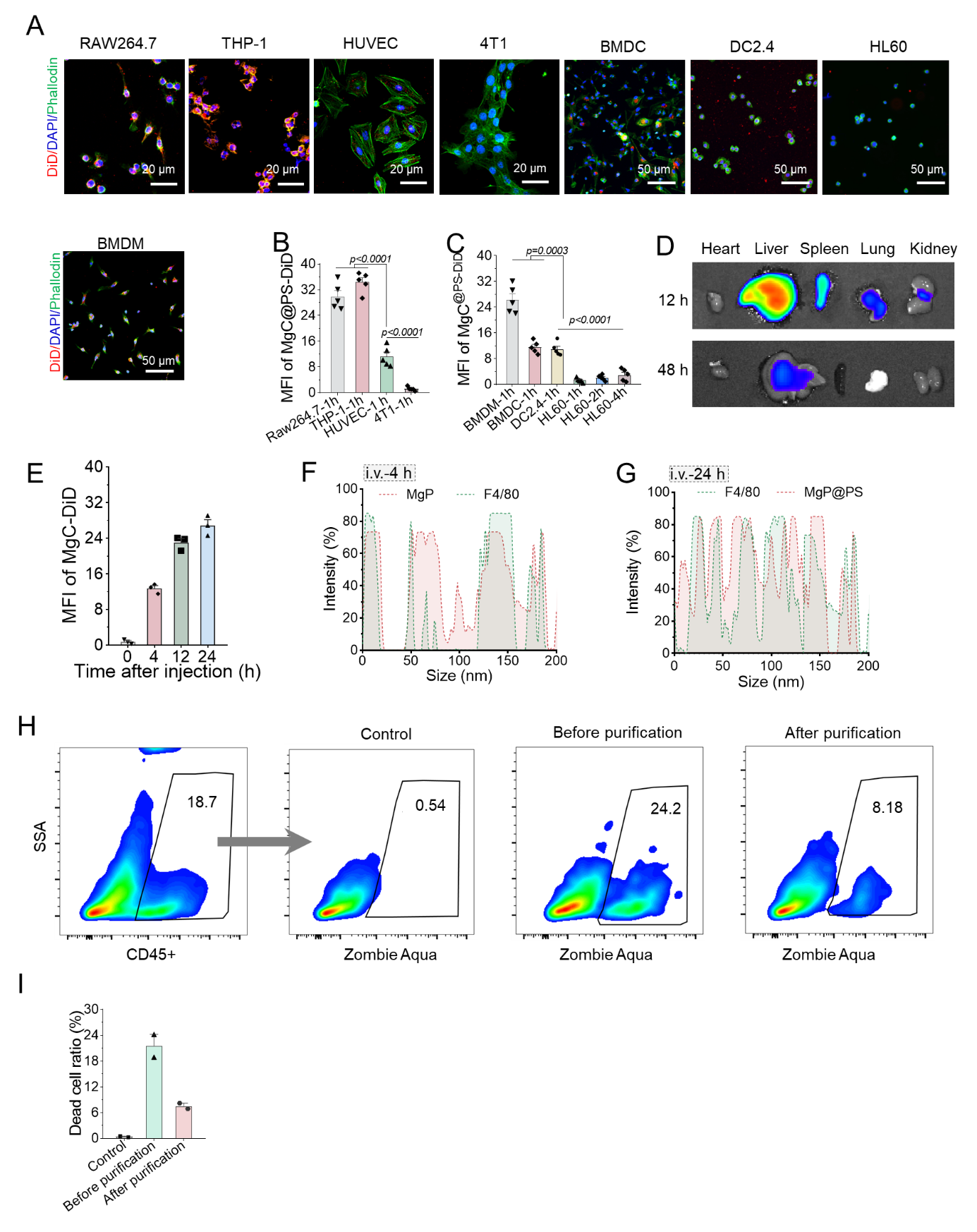


**Figure S2**. (A) Internalization of MgC^@PS^ by different types of cell line, including, Raw 264.7 (mouse monocyte), THP-1 (human monocyte), HUVEC (endothelial cell), 4T1 (tumor cell), BMDC, DC2.4, HL60 cell after 1 h incubation. (B, C) Calculation of mean fluorescence intensity (MFI) of MgC^@PS^ in different cell types according to panel E by imageJ (n = 5). (D) In vivo optical imaging system (IVIS) showing in vivo distribution of MgC^@PS^ in main organs after 12 h, 48 h post intravenous (i.v.) injection. (E) MFI of MgC^@PS-DiD^ of colon tissue sections. (F, G) Fluorescence distribution profile of MgC^@PS/DiD^ and macrophage along the solid white lines. The overlap area indicates the macrophage internalization of MgC^@PS/DiD^.


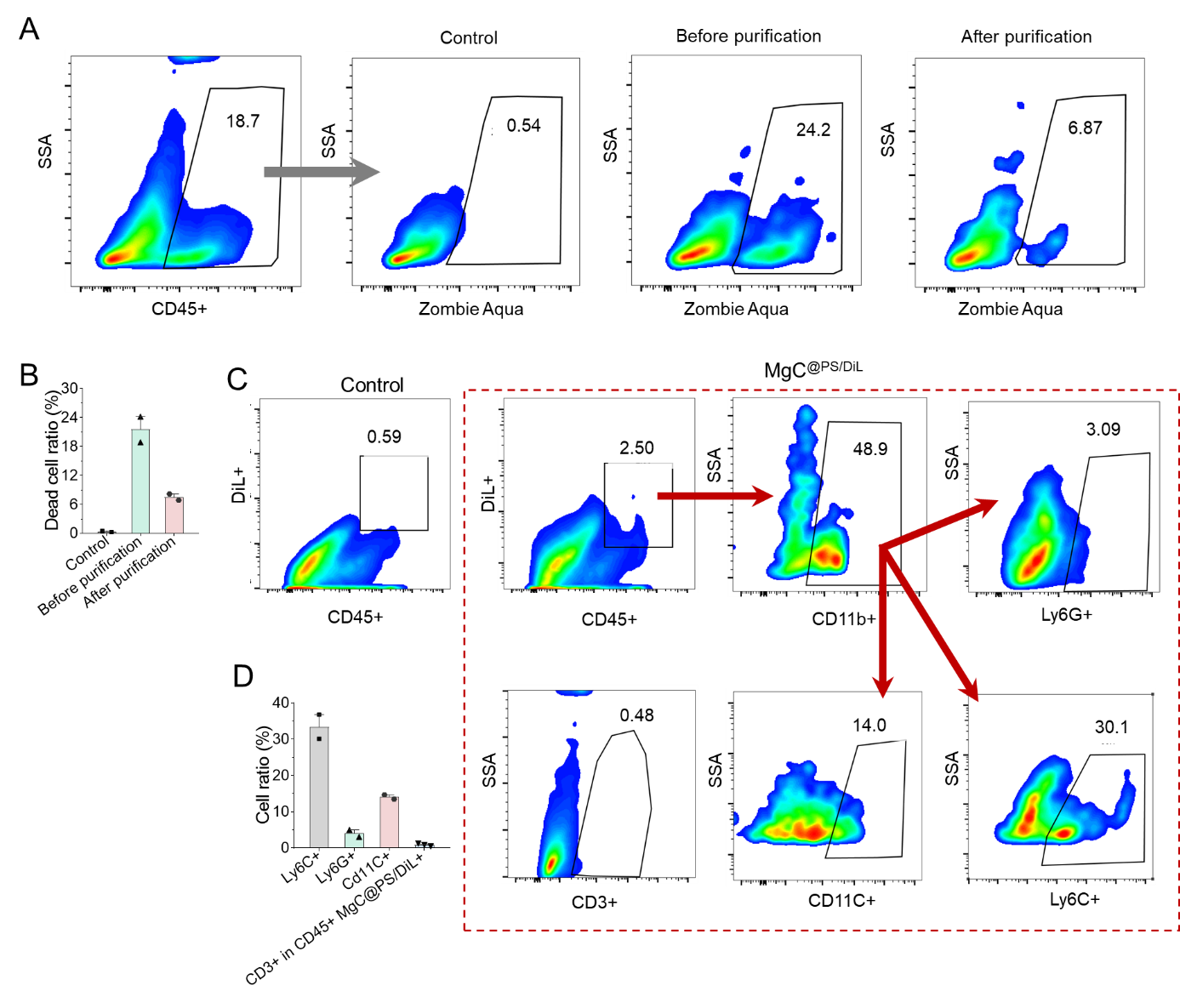


**Figure S3.** (A) Flow cytometry analysis of cells isolated from 3% DSS-pretreated mouse colon tissues, before and after treatment with the Dead Cell Removal Kit. (B) Proportion of dead cells in the flow cytometry-analyzed population following Dead Cell Removal Kit treatment. (C) Flow cytometry analysis of cell types within the MgC@PS/DiL-positive CD45+ cell population; cells were isolated from 3% DSS-pretreated mouse colon tissues. (D) Cell type ratios in the MgC@PS/DiL-positive CD45+ cell population.


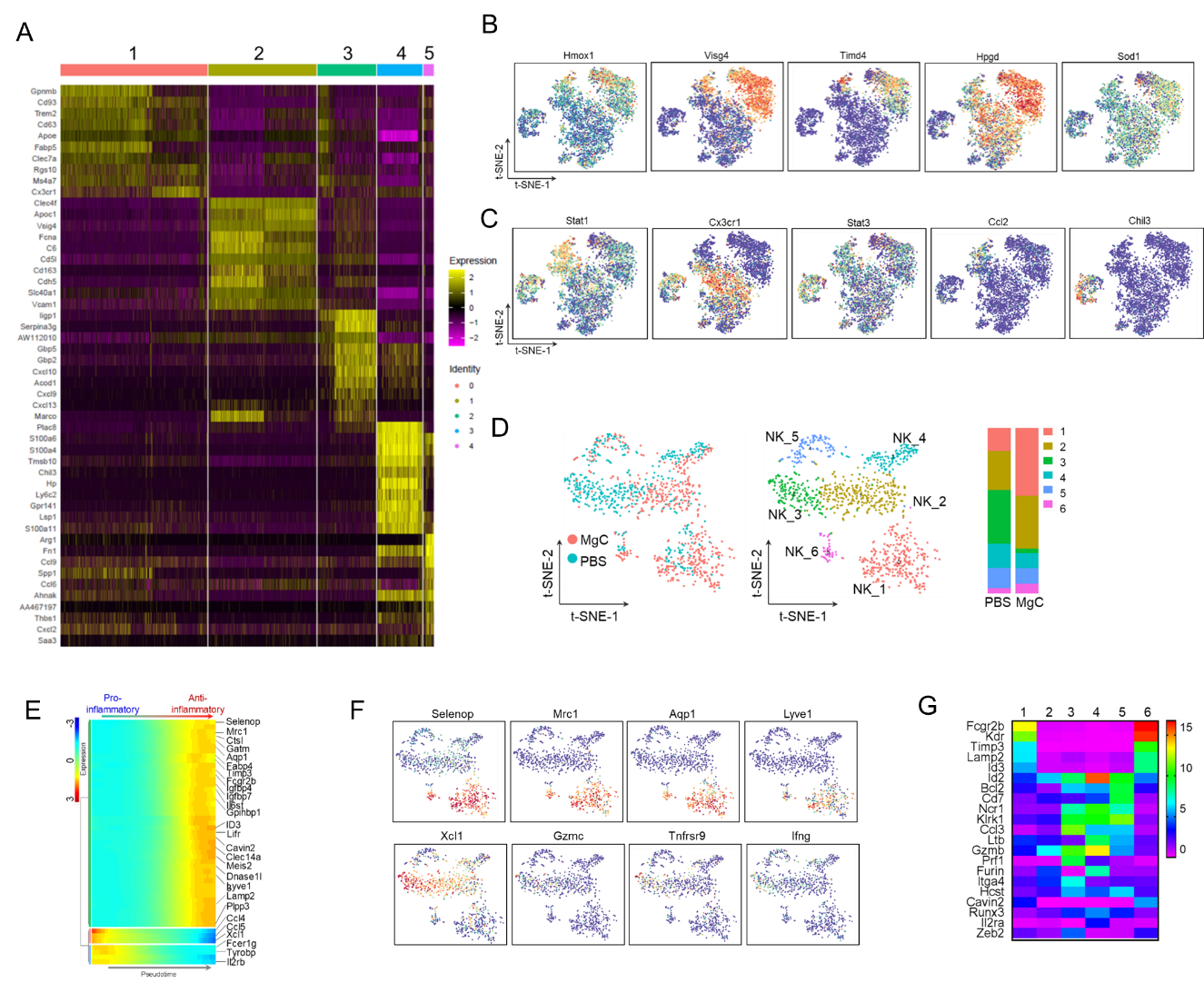


**Figure S4**. (A) Heat map of the top ten differentially expressed genes in each sub-cluster related to Figure 2C. (B) t-SNE plots showing the relative distribution of anti-inflammatory or anti-oxidant genes. (C) t-SNE plots showing the relative distribution of pro-inflammatory genes. (D) Subclusters of six nature killer (NK) cell phenotypes. tSNE visualization of the multiple NK subpopulations from the MgC^@PS^ treatment and control groups identified four subsets. Column chart of the proportions of the five NK subpopulations among all NK in control and MgC^@PS^ treated mice. (E) Heatmap of differential expression genes of NK along pseudotime indicating the change process from pro inflammatory phenotype to anti-inflammatory phenotype. (F) t-SNE plots showing the relative distribution of anti-inflammatory marker (upper level) and pro inflammatory marker (down level). (G) Heatmap of differential expression genes in different subclusters of NKT cells.


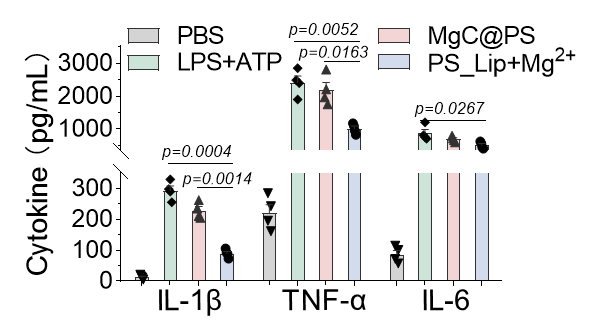


**Figure S5.** ELISA results for pro-inflammatory cytokines (IL-1β, TNF-α, IL-6) secreted by THP-1 cells after LPS treatment, with or without MgC^@PS^ (n = 4)


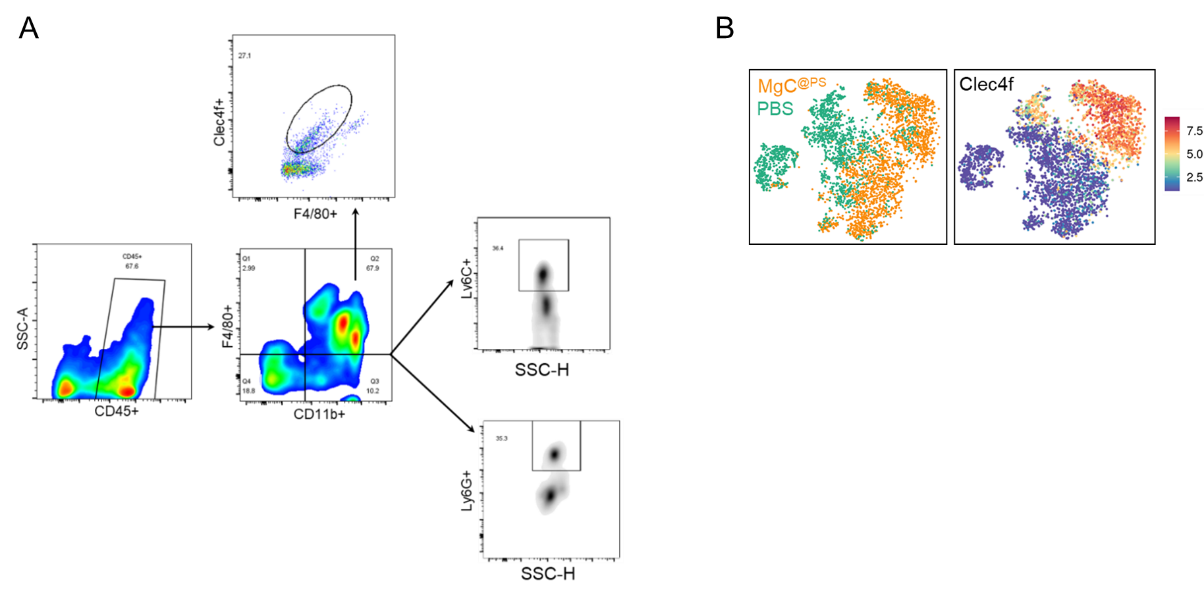


**Figure S6**. (A) Representative flow cytometry gating strategy for Kupffer and infiltrating monocyte analysis in liver tissues from C57BL6 mice after various treatment. (B) t-SNE plots showing the relative distribution of Kupffer cell marker (Clec4f).


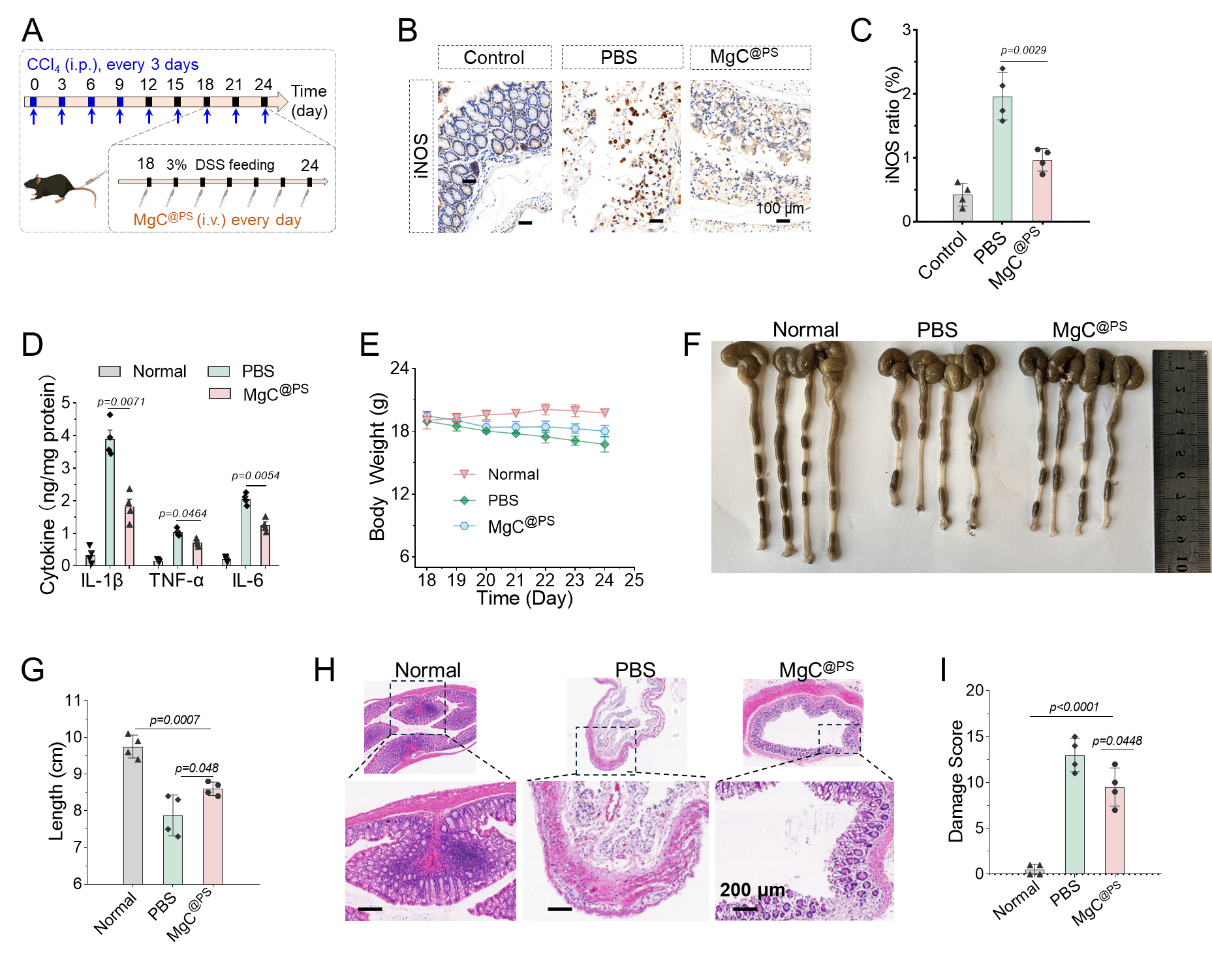


**Figure S7**. (A) Experimental timeline illustrating the induction of liver injury by CCl_s_ and ulcerative colitis by 3%DSS feeding as well as treatment with MgC^@PS^. (B) Immunohistochemical staining showing the iNOS+ pro-inflammatory macrophage distribution in colon tissues. (C) Calculation of iNOS+ macrophage in colon tissues after MgC^@PS^ treatment (n = 4). (D) Pro-inflammatory cytokines in colon tissues after treatment, colon tissues were homogenized and the protein in supernatant was quantified by BCA assay (n = 4). (E) Bodyweight development during treatment (n = 4). (F, G) Photographic images and length calculation of the colon tissues of the controlled and treated mice (n = 4). (H, I) Typical hematoxylin and eosin (H&E) stain images and damages scores of colon tissues after treatments (n = 4).


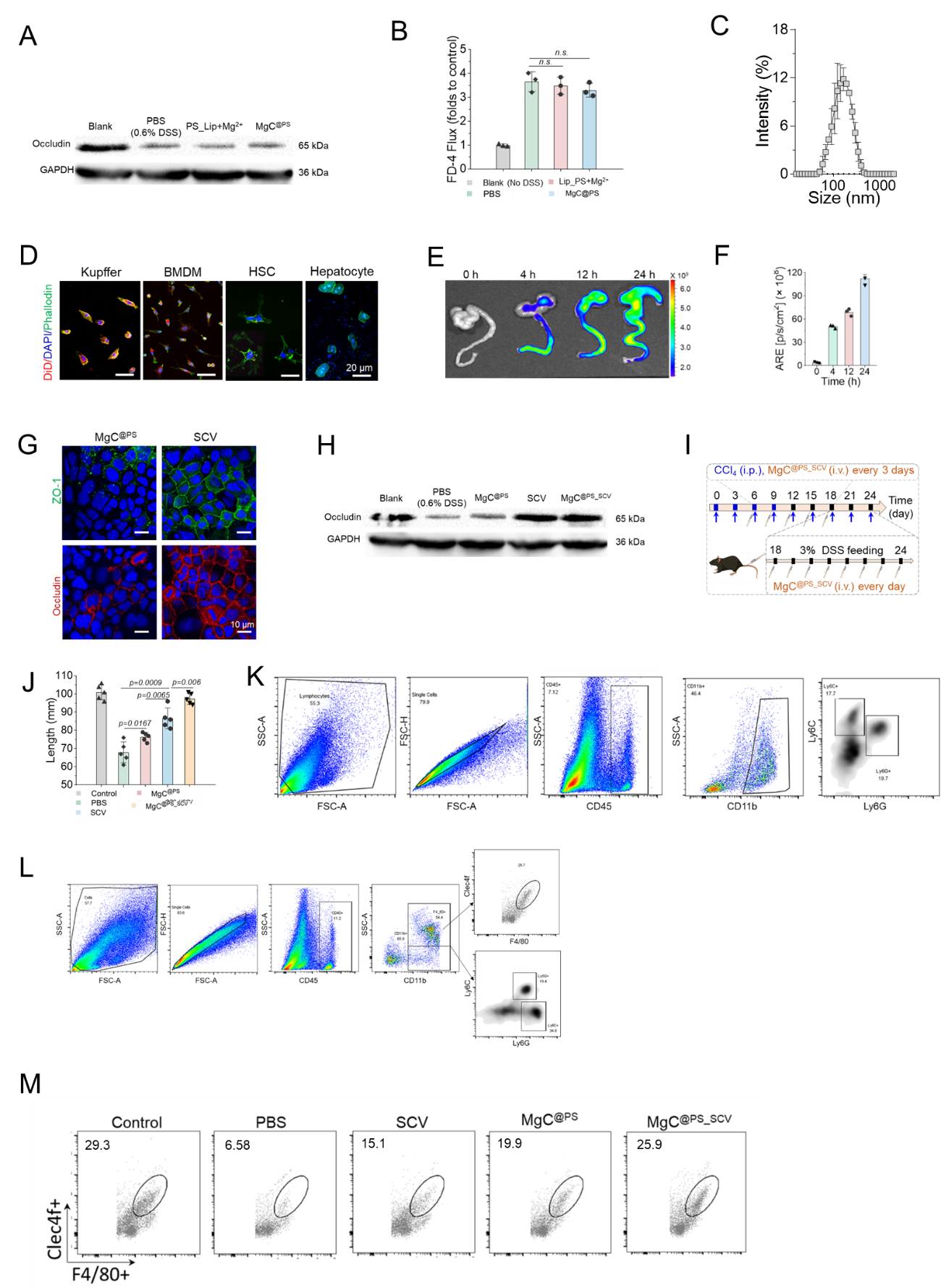


**Figure S8**. (A) Tight junction related protein expression (Occludin) of after treatment of DSS+MgC^@PS^. (B) Fluorescence intensity of FITC in basolateral medium indicating the perfused FITC-dextran (4 kDa) from apical chamber that cross the CaCO-2 monolayer after treatment of DSS+MgC^@PS^. (C) DLS measurements of size distribution MgC^@PS_SCV^. (D) Internalization of MgC^@PS_SCV^ by different types of primary cell types, including, Kupffer cell, BMDM, HSC, Hepatocyte, after 1 h incubation. (E, F) Typical time dependent IVIS images and fluorescence calculation of 3% DSS induced inflammatory mouse colon tissues after MgC^@PS/DiD_SCV^ administration. (G) CLSM images of tight junction protein expression (ZO-1 and Occludin) (related to Figure 5C). (H) Tight junction related protein, occludin, expression of after treatment of DSS+Mg^@PS_SCV^. (I). Experimental timeline illustrating the induction of liver injury and ulcerative colitis by CCl₄ i.p. injection and 3%DSS feeding as well as treatment with MgC^@PS_SCV^. (J) Length calculation of the colon tissues of the controlled and treated mice. (K) Representative flow cytometry gating strategy for infiltrating monocyte analysis in colon tissues from C57BL6 mice after treatment. (L) Representative flow cytometry gating strategy for infiltrating monocyte analysis in liver tissues from C57BL6 mice after treatment. (M) Flow cytometry evaluation of Clec4f+ Kupffer cells of their proportions in liver tissues following treatment.

**Supplementary Table 1.**

Primer list.

|  | Forward | Reverse |
| --- | --- | --- |
| *Il1b*-huamn | CCACAGACCTTCCAGGAGAATG | GTGCAGTTCAGTGATCGTACAGG |
| *Il6*-human | AGACAGCCACTCACCTCTTCAG | TTCTGCCAGTGCCTCTTTGCTG |
| *Tnf*-human | CTCTTCTGCCTGCTGCACTTTG | ATGGGCTACAGGCTTGTCACTC |
| *Acta2*-mouse | TGCTGACAGAGGCACCACTGAA | CAGTTGTACGTCCAGAGGCATAG |
| *Col1a*-mouse | CCTCAGGGTATTGCTGGACAAC | CAGAAGGACCTTGTTTGCCAGG |
| *Gapdh*-human | GTCTCCTCTGACTTCAACAGCG | ACCACCCTGTTGCTGTAGCCAA |
| *Gapdh*-mouse | CATCACTGCCACCCAGAAGACTG | ATGCCAGTGAGCTTCCCGTTCAG |
